# Association of Use of Tourniquets During Total Knee Arthroplasty in the Elderly Patients With Post-operative Pain and Return to Function

**DOI:** 10.3389/fpubh.2022.825408

**Published:** 2022-03-10

**Authors:** Jian Zhao, Xin Dong, Ziru Zhang, Quanyou Gao, Yunfei Zhang, Junlei Song, Shun Niu, Tian Li, Jiying Chen, Fei-Long Wei

**Affiliations:** ^1^Department of Orthopedics, Tangdu Hospital, Fourth Military Medical University, Xi'an, China; ^2^Department of Orthopedics, The First Medical Center, Chinese PLA General Hospital (301 Hospital), Beijing, China; ^3^School of Basic Medicine, Fourth Military Medical University, Xi'an, China

**Keywords:** total knee arthroplasty, tourniquet, function, pain, elder

## Abstract

**Objective:**

During total knee arthroplasty (TKA), tourniquet may negatively impact post-operative functional recovery. This study aimed at investigating the effects of tourniquet on pain and return to function.

**Methods:**

Pubmed, Embase, and Cochrane Library were comprehensively searched for randomized controlled trials (RCTs) published up to February 15th, 2020. Search terms included; total knee arthroplasty, tourniquet, and randomized controlled trial. RCTs evaluating the efficacies of tourniquet during and after operation were selected. Two reviewers independently extracted the data. Effect estimates with 95% CIs were pooled using the random-effects model. Dichotomous data were calculated as relative risks (RR) with 95% confidence intervals (CI). Mean differences (MD) with 95% CI were used to measure the impact of consecutive results. Primary outcomes were the range of motion (ROM) and visual analog scale (VAS) pain scores.

**Results:**

Thirty-three RCTs involving a total of 2,393 patients were included in this study. The mean age is 65.58 years old. Compared to no tourniquet group, the use of a tourniquet resulted in suppressed ROM on the 3rd post-operative day [MD, −4.67; (95% CI, −8.00 to −1.35)] and the 1st post-operative month [MD, −3.18; (95% CI, −5.92 to −0.44)]. Pain increased significantly when using tourniquets on the third day after surgery [MD, 0.39; (95% CI, −0.19 to 0.59)]. Moreover, tourniquets can reduce intra-operative blood loss [MD, −127.67; (95% CI, −186.83 to −68.50)], shorter operation time [MD, −3.73; (95% CI, −5.98 to −1.48)], lower transfusion rate [RR, 0.85; (95% CI, 0.73–1.00)], higher superficial wound infection rates RR, 2.43; [(5% CI, 1.04–5.67)] and higher all complication rates [RR, 1.98; (95% CI, 1.22–3.22)].

**Conclusion:**

Moderate certainty evidence shows that the use of a tourniquet was associated with an increased risk of higher superficial wound infection rates and all complication rates. Therefore, the findings did not support the routine use of a tourniquet during TKA.

## Introduction

Total knee arthroplasty (TKA) is highly effective at relieving joint disease-induced pain and improving joint functions (1–3). However, blood loss during TKA is high, and is estimated to exceed 1,000 ml with 10–38% of patients requiring blood transfusion (4–6). Therefore, to reduce blood loss, tourniquets are routinely used.

Tourniquets, the tourniquet can reduce the overall blood loss and ensure that the surface operation time is clean and bloodless, can reduce total blood loss and create a clean blood-poor surface operation time, thereby achieving a long-term survival rate for cemented TKA components (7, 8). However, clinical applications of tourniquets are associated with some limitations, including delayed quadriceps strength recovery, increased risks of infections, nerve paralysis, and deep vein thrombosis, especially in obese patients (9, 10). Studies (5, 11) have reported that tourniquets can lead to weakened muscles, reduced range of motion (ROM), and increased pain, which may lead to delayed recovery. Li et al. reported that tourniquets can increase the amount of hidden blood loss after surgery (12).

Applications of tourniquets during TKA have been shown to significantly decrease blood loss without exerting adverse effects on early post-operative outcomes (8). Randomized clinical trials (RCT) have shown that the absence of tourniquets does not affect blood loss and bone cement permeability in patients with TKA. Furthermore, less inflammation and better knee functions can be realized without a tourniquet (10). However, there is no consensus regarding the advantages and disadvantages of using tourniquets in TKA. This study aimed at evaluating the effects of tourniquets on functional outcomes, pain and to determine their possible risks during TKA.

## Methods

### Study Protocol

This systematic review of RCTs was performed according to the Cochrane Handbook for Systematic Reviews of Interventions (13) and the Preferred Reporting Items for Systematic Reviews and Meta-analyses (PRISMA) guidelines (14–17).

### Data Sources and Searches

The Cochrane, PROSPERO, Joanna Briggs Institute (JBI), and INPLASY databases were independently searched by two reviewers (J. Z. and T. L.), to avoid duplicates in meta-analysis. Then, we searched electronic databases, including PubMed, Embase and the Cochrane Library (Supplementary Table 1). Searches were performed for publications from database inception to February 15th, 2020. References to relevant comments, editorials, and letters also need to be searched manually.

### Study Selection and Data Extraction

Included Studies were based on the PICOS criteria (Supplementary Table 2).

### Data Extraction

Relevant data was independently collected by two authors (J. Z. and T. L.) based on a well-designed data extraction format that contains the authors' names, publication year, country, participant data, tourniquet pressure, anesthesia method, tourniquet duration, drainage, thrombosis prevention and follow-up.

### Outcomes

Primary outcomes included ROM, pain measured at 3 days, 1, 3, 6, and 12 months post-operatively, and the need for blood transfusion. Secondary outcomes included intra-operative blood loss, post-operative blood loss, measured total blood loss, calculated total blood loss (18), operation time, transfusion, superficial wound infection, deep vein thrombosis (DVT), and all complications (including DVT, infection, revision, wound erythema/ecchymosis among others).

### Quality and Risk-of-Bias Assessment

The Cochrane Collaboration's risk-of-bias assessment tool (19) was used by two reviewers (J. Z., T. L.) to independently evaluate the included studies for potential bias (Supplementary Table 3). Disagreements between the two investigators were resolved by involving a third investigator (F-L. W.). We used the Cochrane risk of bias method to assess bias assessment (20, 21). If there are 4 or more studies per comparison, the funnel asymmetric distribution was used to estimate publication bias (22). Two reviewers (J. Z., T. L.) independently used the GRADE component (23) to categorize the quality and strength of the evidence as high, moderate, low, and very low for the ROM, pain, superficial wound infection rates and all complication rates.

### Data Synthesis and Statistical Analysis

We used STATA 16.0 (Stata Corp, College Station, TX, USA) to analyze data. Data pooling was done using a random-effects model (24). Dichotomous data were evaluated by relative risks (RR) with 95% confidence intervals (CI). Mean differences (MD) with 95% CI were used to weigh effect sizes for continuous outcomes. A forest plot was used to assess effect sizes. The weight of the included study depends on the value of the event in the treatment group, the event in the control group, and the size of the entire sample. *P* ≤ 0.05 indicates that the difference is statistically significant. Statistical heterogeneity among summary data were evaluated using the chi-square test and *I*^2^ statistic. If the chi-square test showed *p* < 0.10 and *I*^2^ > 50%, data showed high heterogeneity. A subgroup analysis was conducted based on anesthesia, Tourniquet duration, drainage, thromboprophylaxis. Because these variables are categorical variables, we did not do meta regression.

## Results

### Studies Retrieved

During our literature search, collation and analysis, no duplicate meta-analysis topics were found in the databases. The PRISMA flow chart of the selection process retrieved a total of 440 results, of which 245 (56.68%) remained after removal of duplicates (Supplementary Figure 1). Six relevant studies were added. After title/abstract curation, a total of 193 records were excluded, with 58 articles remaining. Then, the full text was read and 33 eligible RCTs (34 articles) were enrolled for final synthesis.

### Study Characteristics

Thirty-three RCTs involving a total of 2,393 patients participated in this meta-analysis (Supplementary Table 4) (5, 6, 8, 10, 12, 25–52). The mean age is 65.58 years old. These studies come from North America, Europe, Asia, and Latin America and had been published between 1995 and 2021. Based on our defined outcomes, 12 reported ROM outcomes (5, 6, 8, 10, 12, 35, 44, 46–49, 52); 10 reported pain outcomes (6, 8, 10, 39, 44, 46, 47, 49–51); 14 reported intra-operative blood loss outcomes (5, 6, 10, 12, 28, 30, 32, 37–39, 41, 43, 48); 11 reported post-operative blood loss outcomes (6, 26, 28, 30, 32, 33, 37–40, 48); 9 reported measured total blood loss outcomes (5, 6, 10, 28, 30, 31, 34, 37, 39); 9 reported calculated total blood loss outcomes (8, 30, 32, 39, 41–43, 48, 52); 20 reported operation time outcomes (5, 6, 8, 10, 12, 28, 30, 32, 33, 35, 38, 39, 42–44, 46–48, 51, 52); 16 reported transfusion outcomes (5, 6, 8, 10, 26, 29, 30, 32, 35, 36, 39, 42, 43, 48, 50, 52); 16 reported DVT outcomes (5, 6, 8, 25–27, 32, 33, 35, 37, 48, 51, 52); 8 reported superficial wound infection outcomes (5, 6, 8, 25, 30, 48, 49, 51); while 15 reported the outcomes for all complications (5, 6, 8, 25, 30–33, 35, 39, 42, 44, 48, 49, 51).

### Risk of Bias Assessments

Supplementary Figures 2, 3 summarizes the assessment of the risk of bias of selected articles. Four studies were found to have a high risk for randomization sequence generation (10, 37, 38, 50), with 8 not providing this information (25, 26, 28, 30, 31, 33, 36, 43); 5 studies showed a high risk in concealing allocation (25, 26, 31, 33, 38), with 8 not providing this information (12, 27, 28, 36, 37, 39, 50, 51). Due to the nature of intervention, it is not possible to blind participants and therapists in any study. Thirteen of these studies included the objective results of blindly evaluating assessors (5, 6, 8, 32, 41, 42, 45, 46, 48, 49, 52). No study showed a high risk in selective outcome reporting.

### Primary Outcomes

#### ROM

**Figure 1:**
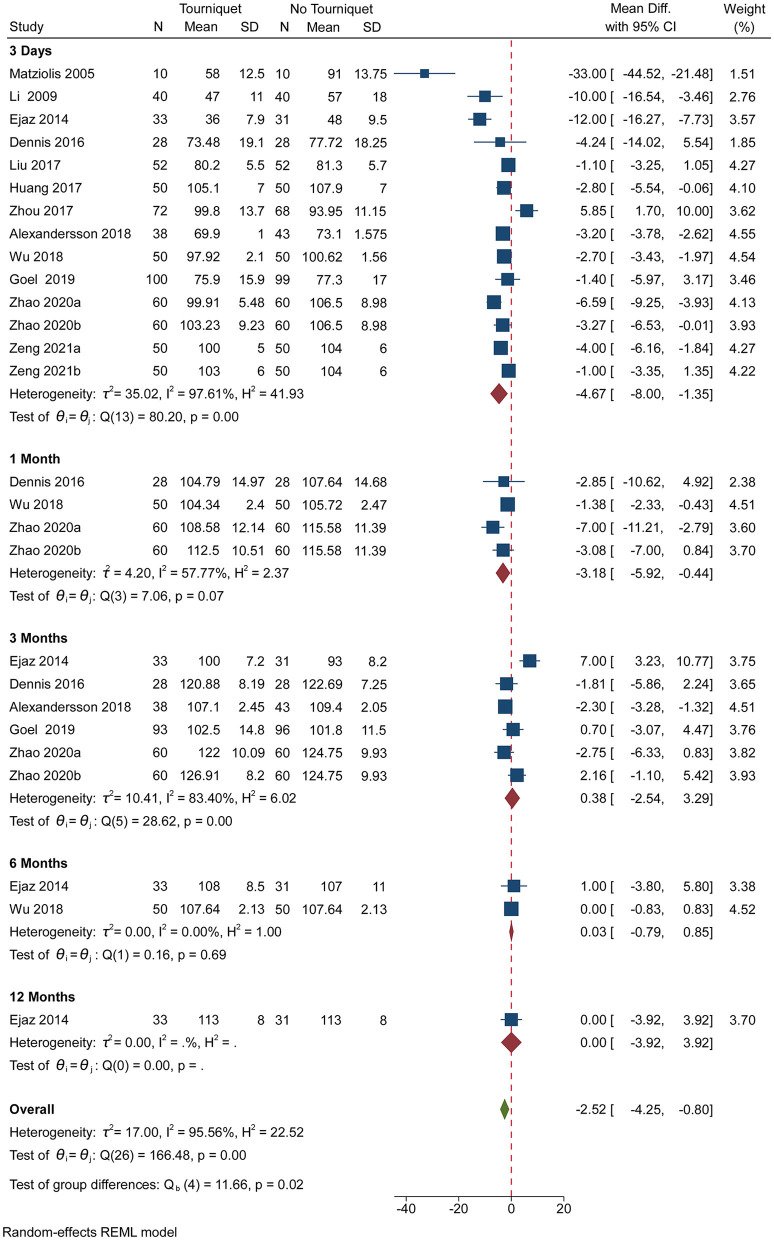
Forest plot comparing ROM outcomes in no tourniquet and tourniquet groups.

Pooled analysis of 12 studies showed significantly suppressed ROM When a tourniquet is put on the 3rd day after the operation [MD, −4.67; (95% CI, −8.00 to −1.35)] and the 1st post-operative month [MD, −3.18; (95% CI, −5.92 to −0.44)] (Figure 1). However, applications of a tourniquet did not have a significant impact on ROM on the 3rd, 6th and 12th post-operative months (Figure 1). More than 50% heterogeneity was found in studies reporting ROM on the 3rd post-operative day, the 1st and 3rd post-operative month (Figure 1). Supplementary Figure 4, a contour-enhanced funnel plot, showed significant deviations in the publication. Subgroup analysis revealed that anesthesia, tourniquet duration, and drainage did not affect ROM, whereas thromboprophylaxis had effect on ROM on the 3rd post-operative day (*p* = 0.00) (Supplementary Figure 5). Subgroup analysis showed that drainage affected ROM on the 1st post-operative month (*p* = 0.03; Supplementary Figure 6) while thromboprophylaxis affected ROM on the 3rd post-operative month (*p* = 0.00; Supplementary Figure 7). Based on GRADE assessment, moderate-quality evidence suggests that the use of a tourniquet resulted in suppressed ROM on the 3rd post-operative day and the 1st post-operative month.

#### Pain

**Figure 2:**
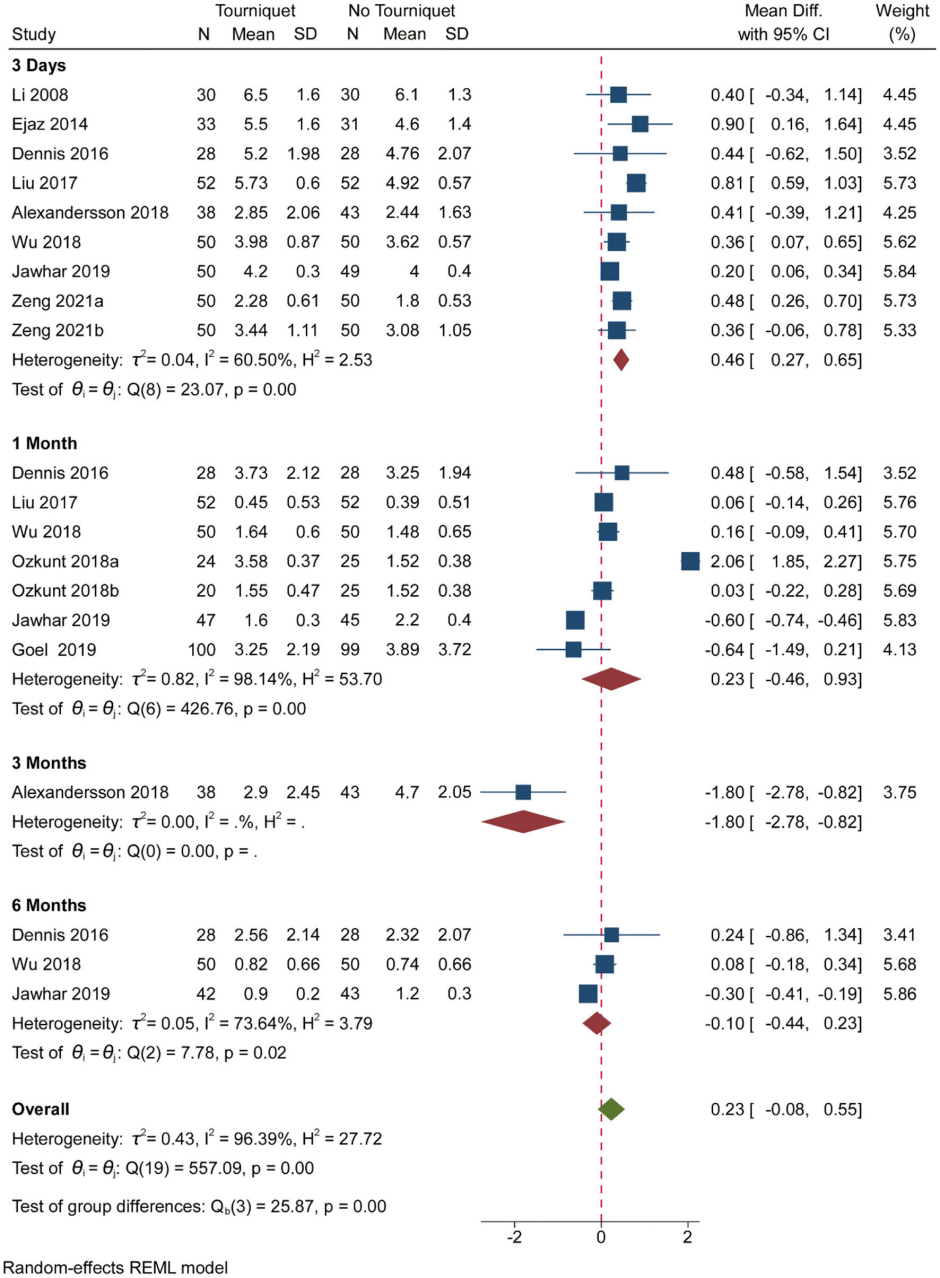
Forest plot comparing pain outcomes in no tourniquet and tourniquet groups.

Pooled analysis of 10 studies showed that pain was significantly increased when a tourniquet was used on the 3rd post-operative day [MD, 0.46; (95% CI, 0.27–0.65); Figure 2]. Pain was significantly reduced when a tourniquet was applied on the 3rd post-operative day [MD, −1.80; (95% CI, 2.78 to −0.82); Figure 2]. However, tourniquets had no meaningful impact on pain in the 1st, 3rd, 6th, and 12th post-operative months (Figure 2). Supplementary Figure 8, a contour-enhanced funnel plot, did reveal significant publication bias. More than 50% heterogeneity was found in studies reporting pain on the 3rd postoperative day and the 1st post-operative month (Figure 2). Subgroup analysis showed that anesthesia, tourniquet duration, drainage, and thromboprophylaxis did not affect pain outcomes (Supplementary Figures 9, 10). Based on GRADE assessment, moderate-quality evidence suggests that pain was significantly increased when a tourniquet was used on the 3rd post-operative day.

### Secondary Outcomes

#### Blood Loss

**Figure 3:**
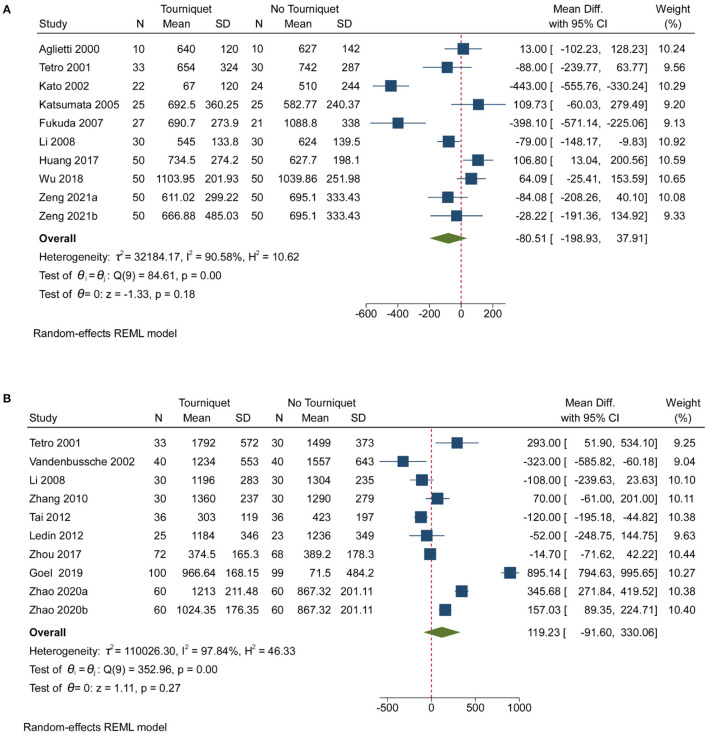
Forest plot for measured total blood loss and calculated total blood loss. **(A)**. Forest plot comparing measured total blood loss in no tourniquet and tourniquet groups; **(B)**. Forest plot comparing calculated total blood loss in no tourniquet and tourniquet groups.

Pooled analysis of 14 studies showed that the use of a tourniquet resulted in low intra-operative blood loss [MD, −127.67; (95% CI, −186.83 to −68.50); Supplementary Figure 11]. Heterogeneity (99.12%) was found in studies reporting on intra-operative blood loss (Supplementary Figure 11). Supplementary Figure 12, contour-enhanced funnel plot, showed significant deviations in the publication. Pooled analysis revealed that tourniquets had no meaningful impact on post-operative blood loss, measured total blood loss and calculated total blood loss (Figure 3 and Supplementary Figure 11). Contour-enhanced funnel plots (Supplementary Figures 14, 16, 18) showed significant deviations in the publication. More than 50% heterogeneity was found in studies reporting post-operative blood loss, measured total blood loss and calculated total blood loss (Supplementary Figure 11 and Figure 3). Subgroup analyses showed that thromboprophylaxis affected intra-operative blood loss (*p* = 0.00; Supplementary Figure 13). However, anesthesia, tourniquet duration, drainage, and thromboprophylaxis did not affect post-operative blood loss or measured total blood loss (Supplementary Figures 15, 17). In addition, anesthesia, tourniquet duration, drainage, and thromboprophylaxis affected the calculated total blood loss (*p* = 0.02, 0.01, and 0.03, respectively; Supplementary Figure 19).

#### Operation Time

Pooled analysis of 20 studies showed that tourniquets were associated with a shorter operation time [MD, −3.73; (95% CI, −5.98 to −1.48); Supplementary Figure 20]. Supplementary Figure 21, a contour-enhanced funnel plot, showed significant deviations in the publication. An 84.83% heterogeneity was found across studies reporting on operation time (Supplementary Figure 20). Subgroup analysis revealed that drainage affected operation time (*p* = 0.00; Supplementary Figure 22).

#### Complications

**Figure 4:**
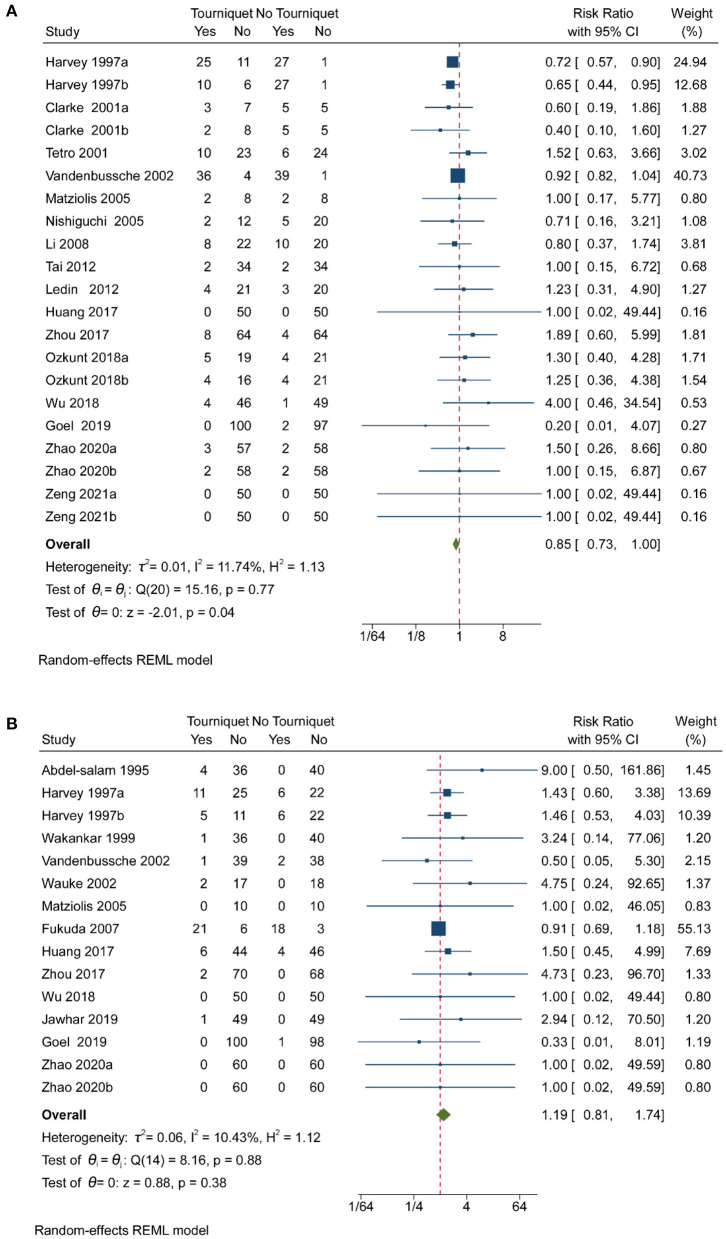
Forest plot for transfusion rate and DVT. **(A)** Forest plot comparing transfusion rates between no tourniquet and tourniquet groups; **(B)** Forest plot comparing DVT outcomes between no tourniquet and tourniquet groups.

**Figure 5:**
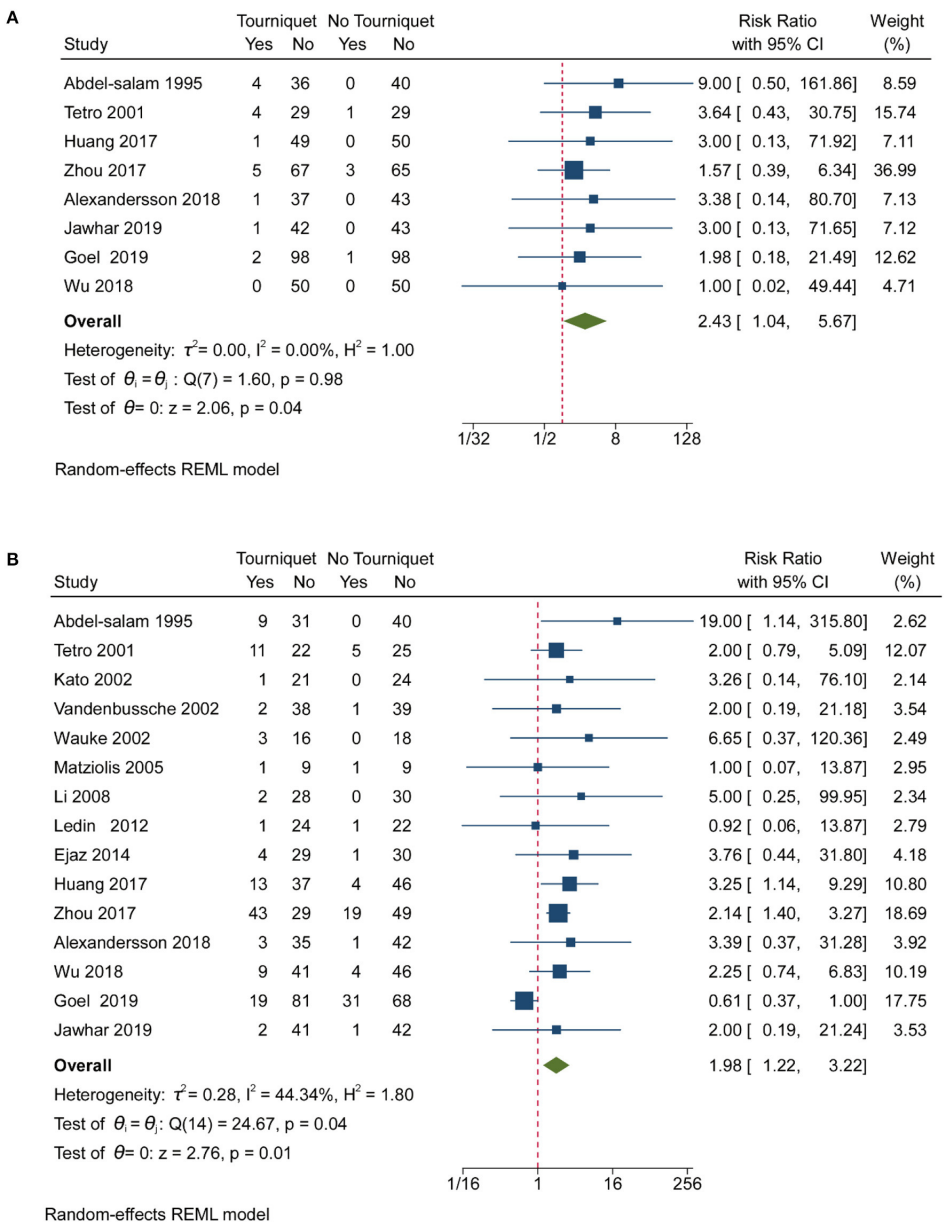
Forest plot for superficial wound infection and all complication rates. **(A)** Forest plot comparing superficial wound infection rates between the no tourniquet and tourniquet groups; **(B)** Forest plot comparing all complication rates between the no tourniquet and tourniquet groups.

Pooled analysis of 16 studies showed that tourniquets are associated with low transfusion rates [RR, 0.85; (95% CI, 0.73–1.00); Figure 4A]. Less than 25% heterogeneity was found in studies reporting on transfusion (Figure 4A). Pooled analysis of 16 studies showed that tourniquets had no meaningful impact on DVT (Figure 4B). A 10.43% heterogeneity was found across studies reporting on transfusion (Figure 4B). A pooled analysis of 8 studies showed that tourniquets are associated with higher superficial wound infection rates [RR, 2.43; (95% CI, 1.04–5.67); Figure 5A]. A 0% heterogeneity was found across studies reporting on superficial wound infection (Figure 5A). In addition, pooled analysis of 15 studies showed that tourniquets are associated with higher all complication rates [RR, 1.98; (95% CI, 1.22–3.22); Figure 5B]. Less than 50% heterogeneity was found across studies reporting on transfusion (Figure 5B). Contour-enhanced funnel plots (Supplementary Figures 23–26) did not show significant publication bias. Pooled analysis of 7 studies showed that tourniquets have no association with pulmonary embolism rate [RR, 1.71; (95% CI, 0.49–6.00); Supplementary Figure 27]. Contour-enhanced funnel plots (Supplementary Figure 28) showed no significant publication bias. Pooled analysis of 7 studies showed that tourniquets have no association with pulmonary embolism rate [RR, 1.71; (95% CI, 0.49–6.00); Supplementary Figure 27]. Contour-enhanced funnel plots (Supplementary Figure 28) showed no significant publication bias. Based on GRADE assessment, moderate-quality evidence suggests that the use of a tourniquet was with an increased risk of higher superficial wound infection rates and all complication rates.

## Discussion

Applications of tourniquets in TKA are not supported by sufficient data. Evidence regarding the effects of tourniquets on perioperative blood loss, post-operative function, and pain is not conclusive. We found that tourniquets are associated with increased post-operative pain and/or diminished short term functional outcomes. However, these effects disappeared after 1 month. Our findings do not support the use of tourniquets during TKA. However, differences in outcomes were small and do not have much clinical significance (53), so interpretation of the conclusion should be cautious.

Guler et al. reported that clinical applications of tourniquets during TKA led to a 20% reduction in quadriceps volume after surgery, when measured against contralateral limb at 1-month of follow-up. There were no differences between limbs on which tourniquets were not applied (54). Dennis et al. reported simultaneous bilateral TKA, in which a tourniquet was used on one knee, and muscle weakness in the tourniquet group lasted until 3 months after surgery (46). However, Goel et al. showed that there were no clinical differences between patients who had inflated tourniquets and those who did not, by assessing functions of treated limbs (8). We found that tourniquets slow down patients' functional recoveries and increases ischemia-associated pain. Thigh pain is common when tourniquets are used in early post-operative periods (55). Pain is unfavorable and hinders joint function recovery. The ROM is significantly decreased when tourniquets are used in TKA. Our findings are consistent with those of previous trials (5, 6, 10, 52, 56). However, differences in ROM reported in this study were within the error of goniometer, which ranges from 4 to 8 degrees, therefore, these differences might not be clinically relevant.

Total knee arthroplasty is associated with large amounts of perioperative blood loss; reduced bleeding reduces transfusion incidences. Clinical applications of tranexamic acid, hypotension-controlled anesthesia, and tourniquets are widely used in surgery (52, 57). An RCT showed that tourniquets can reduce calculated blood loss during the perioperative period (8). Moreover, it has been reported that applications of tourniquets in TKA increase total blood loss (12, 58). We found that tourniquets do not affect calculated blood loss. However, >50% heterogeneity was found across studies that reported calculated total blood loss. Subgroup analysis showed that anesthesia and thromboprophylaxis affect calculated total blood loss. A previous meta-analysis revealed that early tourniquet release is associated with greater perioperative blood loss, compared to tourniquet release after wound closure (59). The reason for this difference might be because we included more updated RCTs. The results showed that tourniquets are associated with decreased intra-operative blood loss, which can improve the surgical field of vision. Therefore, operation times are shorter when tourniquets are used.

Although there was no difference in calculated total blood loss between the two groups, tourniquets were associated with lower transfusion rates. Mori et al. reported that tourniquets are associated with a greater risk of DVT, following TKA (60). Our study found that there are no differences in DVT between the two groups, consistent with a previous meta-analysis (61). Long-term effects of tourniquets with regarding to post-operative complications have not been clearly established. A matched cohort study showed that increased tourniquet times are associated with increased 30-day readmission rates (62). The longer a tourniquet is used, the higher the risk of wound complications (63). In this study, superficial wound infection rates and all complications were found to be higher in the tourniquet group. Goel et al. found that the total number of complications was high in the no-tourniquet group, however, differences in complication rates were not significant (8). Our results are consistent with those of a previous study (61).

## Strength and Limitations

This study has some strengths, first, this meta-analysis was performed by a professional team including a Cochrane member. Second, the methods were inclusive and transparent, including all software and website sources. Third, analyses were refined on Patient, Intervention, Control, Outcomes, and Study design (PICOS principle). In addition, the study included 12 outcomes to comprehensively evaluate the effects of tourniquets.

However, it is associated with some limitations. First, PubMed, Embase, and Cochrane Library were searched whereas other databases such as Web of Science, was not. PubMed, Embase, and Cochrane Library include almost all databases and a retrieval strategy was formulated (64). Second, many studies did not clarify the duration of tourniquet use. In addition, high heterogeneity of blood loss is a disadvantage that affects result reliability. Different surgical techniques and different measurement methods may lead to this heterogeneity.

## Conclusions

This meta-analysis provides insights into evidence-based medicine currently approved by the Cochrane Collaboration (65). Our findings do not support routine use of tourniquets during TKA, as inflating the tourniquet was associated with more pain, slower functional recovery and more complications. However, this conclusion should be interpreted cautiously, considering the small differences in outcomes.

## Data Availability Statement

The original contributions presented in the study are included in the article/Supplementary Material, further inquiries can be directed to the corresponding author/s.

## Author Contributions

F-LW and JC: had full access to all the data in this study and they take responsibility for data integrity and accuracy of analysis. JC, F-LW, JZ, TL, XD, ZZ, and QG: concept and design. JC, F-LW, JZ, TL, XD, ZZ, QG, YZ, JS, and SN: acquisition, analysis, and interpretation of data. JZ, F-LW, and JC: drafting of the manuscript. TL, JZ, and F-LW: statistical analysis. JC, F-LW, JZ, TL, XD, ZZ, and QG: administrative, technical, or material support. JC, F-LW, JZ, and TL: supervision. All authors: critical revision of the manuscript for important intellectual content. All authors contributed to the article and approved the submitted version.

## Funding

This study was supported by Tangdu Hospital Seed Talent Program (F-LW) and Natural Science Basic Research Plan in Shaanxi Province of China (No. 2019JM-265). The funding body had no role in the design of the study, data collection, analysis, interpretation, or in writing the manuscript.

## Conflict of Interest

The authors declare that the research was conducted in the absence of any commercial or financial relationships that could be construed as a potential conflict of interest.

## Publisher's Note

All claims expressed in this article are solely those of the authors and do not necessarily represent those of their affiliated organizations, or those of the publisher, the editors and the reviewers. Any product that may be evaluated in this article, or claim that may be made by its manufacturer, is not guaranteed or endorsed by the publisher.

## References

[1] Wu Y Zeng Y Bao X Xiong H Hu Q Li M . Comparison of mini-subvastus approach versus medial parapatellar approach in primary total knee arthroplasty. Int J Surg. (2018) 57:15–21. 10.1016/j.ijsu.2018.07.007 30056128

[2] Chen Z Shen Z Ye X Xu Y Liu J Shi X . Acupuncture for rehabilitation after total knee arthroplasty: a systematic review and meta-analysis of randomized controlled trials. Front Med. (2020) 7:602564. 10.3389/fmed.2020.602564 33553202 PMC7856874

[3] Peng L Wang K Zeng Y Wu Y Si H Shen B. Effect of neuromuscular electrical stimulation after total knee arthroplasty: a systematic review and meta-analysis of randomized controlled trials. Front Med. (2021) 8:779019. 10.3389/fmed.2021.779019 34926522 PMC8677678

[4] Park JH Rasouli MR Mortazavi SM Tokarski AT Maltenfort MG Parvizi J. Predictors of perioperative blood loss in total joint arthroplasty. J Bone Joint Surg Am Vol. (2013) 95:1777–83. 10.2106/JBJS.L.01335 24088970

[5] Huang Z Xie X Li L Huang Q Ma J Shen B . Intravenous and topical tranexamic acid alone are superior to tourniquet use for primary total knee arthroplasty: a prospective, randomized controlled trial [comparative study; journal article; randomized controlled trial]. J Bone Joint Surg Am Vol. (2017) 99:2053–61. 10.2106/JBJS.16.01525 29257010

[6] Wu Y Lu X Ma Y Zeng Y Xiong H Bao X . Efficacy and safety of limb position on blood loss and range of motion after total knee arthroplasty without tourniquet: a randomized clinical trial [journal article; randomized controlled trial]. Int J Surg. (2018) 60:182–7. 10.1016/j.ijsu.2018.11.008 30468901

[7] Cai DF Fan QH Zhong HH Peng S Song H. The effects of tourniquet use on blood loss in primary total knee arthroplasty for patients with osteoarthritis: a meta-analysis. J Orthop Surg Res. (2019) 14:348. 10.1186/s13018-019-1422-4 31703706 PMC6839231

[8] Goel R Rondon AJ Sydnor K Blevins K O'Malley M Purtill JJ . Tourniquet use does not affect functional outcomes or pain after total knee arthroplasty: a prospective, double-blinded, randomized controlled trial. J Bone Joint Surg Am Vol. (2019) 101:1821–8. 10.2106/JBJS.19.00146 31626006

[9] Kerkhoffs GM Servien E Dunn W Dahm D Bramer JA Haverkamp D. The influence of obesity on the complication rate and outcome of total knee arthroplasty: a meta-analysis and systematic literature review. J Bone Joint Surg Am Vol. (2012) 94:1839–44. 10.2106/JBJS.K.00820 23079875 PMC3489068

[10] Zeng Y Li Y Si H Wu Y Li M Liu Y . Effects of tourniquet use on clinical outcomes and cement penetration in TKA when tranexamic acid administrated: a randomized controlled trial. BMC Musculoskel Disord. (2021) 22:126. 10.1186/s12891-021-03968-5 33517881 PMC7847577

[11] Harsten A Bandholm T Kehlet H Toksvig-Larsen S. Tourniquet versus no tourniquet on knee-extension strength early after fast-track total knee arthroplasty; a randomized controlled trial. Knee. (2015) 22:126–30. 10.1016/j.knee.2014.12.010 25648580

[12] Li B Wen Y Wu H Qian Q Lin X Zhao H. The effect of tourniquet use on hidden blood loss in total knee arthroplasty [Journal Article; Randomized Controlled Trial]. Int Orthop. (2009) 33:1263–8. 10.1007/s00264-008-0647-3 18751703 PMC2899119

[13] Higgins JPT GS, eds. Cochrane Handbook for Systematic Reviews of Interventions, Version 5.1.0. (2011). Available online at: https://handbook-5--1.cochrane.org/ (accessed March 27, 2019).

[14] Liberati A Altman DG Tetzlaff J Mulrow C Gøtzsche PC Ioannidis JP . The PRISMA statement for reporting systematic reviews and meta-analyses of studies that evaluate healthcare interventions: explanation and elaboration. BMJ. (2009) 339:b2700. 10.1136/bmj.b2700 19622552 PMC2714672

[15] Wei FL Zhou CP Liu R Zhu KL Du MR Gao HR . Management for lumbar spinal stenosis: a network meta-analysis and systematic review. Int J Surg. (2021) 85:19–28. 10.1016/j.ijsu.2020.11.014 33253898

[16] Zhang F Wang K Du P Yang W He Y Li T . Risk of stroke in cancer survivors: a meta-analysis of population-based cohort studies. Neurology. (2021) 96:e513–26. 10.1212/WNL.0000000000011264 33277416

[17] Li T Providencia R Mu N Yin Y Chen M Wang Y . Association of metformin monotherapy or combined therapy with cardiovascular risks in patients with type 2 diabetes mellitus. Cardiovasc Diabetol. (2021) 20:30. 10.1186/s12933-020-01202-5 33516224 PMC7847575

[18] Gross JB. Estimating allowable blood loss: corrected for dilution. Anesthesiology. (1983) 58:277–80. 10.1097/00000542-198303000-00016 6829965

[19] Higgins JP Altman DG Gøtzsche PC Jüni P Moher D Oxman AD . The Cochrane Collaboration's tool for assessing risk of bias in randomised trials. BMJ. (2011) 343:d5928. 10.1136/bmj.d5928 22008217 PMC3196245

[20] Wei FL Zhou CP Zhu KL Du MR Liu Y Heng W . Comparison of different operative approaches for lumbar disc herniation: a network meta-analysis and systematic review. Pain Physician. (2021) 24:E381–92. 10.36076/ppj.2021.24.E381 34213864

[21] Ma LL Wang YY Yang ZH Huang D Weng H Zeng XT. Methodological quality (risk of bias) assessment tools for primary and secondary medical studies: what are they and which is better? Military Med Res. (2020) 7:7. 10.1186/s40779-020-00238-8 32111253 PMC7049186

[22] Sterne JA Sutton AJ Ioannidis JP Terrin N Jones DR Lau J . Recommendations for examining and interpreting funnel plot asymmetry in meta-analyses of randomised controlled trials. BMJ. (2011) 343:d4002. 10.1136/bmj.d4002 21784880

[23] Meader N King K Llewellyn A Norman G Brown J Rodgers M . A checklist designed to aid consistency and reproducibility of GRADE assessments: development and pilot validation. Syst Rev. (2014) 3:82. 10.1186/2046-4053-3-82 25056145 PMC4124503

[24] DerSimonian R Laird N. Meta-analysis in clinical trials. Control Clin Trials. (1986) 7:177–88. 10.1016/0197-2456(86)90046-2 3802833

[25] Abdel-Salam A Eyres KS. Effects of tourniquet during total knee arthroplasty. A prospective randomised study. J Bone Joint Surg Br Vol. (1995) 77:250–3. 10.1302/0301-620X.77B2.7706340 7706340

[26] Harvey EJ Leclerc J Brooks CE Burke DL. Effect of tourniquet use on blood loss and incidence of deep vein thrombosis in total knee arthroplasty. J Arthroplasty. (1997) 12:291–6. 10.1016/S0883-5403(97)90025-5 9113543

[27] Wakankar HM Nicholl JE Koka R D'Arcy JC. The tourniquet in total knee arthroplasty. A prospective, randomised study [Article]. J Bone Joint Surg Br Vol. (1999) 81:30–3. 10.1302/0301-620X.81B1.0810030 10067997

[28] Aglietti P Baldini A Vena LM Abbate R Fedi S Falciani M. Effect of tourniquet use on activation of coagulation in total knee replacement [Clinical Trial; Journal Article; Randomized Controlled Trial]. Clin Orthop Relat Res. (2000) 169–77. 10.1097/00003086-200002000-00021 10693564

[29] Clarke MT Longstaff L Edwards D Rushton N. Tourniquet-induced wound hypoxia after total knee replacement [Clinical Trial; Comparative Study; Journal Article; Randomized Controlled Trial; Research Support, Non-U.S. Gov't]. J Bone Joint Surg Br Vol. (2001) 83:40–4. 10.1302/0301-620X.83B1.0830040 11245536

[30] Tetro AM Rudan JF. The effects of a pneumatic tourniquet on blood loss in total knee arthroplasty [Clinical Trial; Journal Article; Randomized Controlled Trial]. Can J Surg. (2001) 44:33–8. 11220796 PMC3695181

[31] Kato N Nakanishi K Yoshino S Ogawa R. Abnormal echogenic findings detected by transesophageal echocardiography and cardiorespiratory impairment during total knee arthroplasty with tourniquet. Anesthesiology. (2002) 97:1123–8. 10.1097/00000542-200211000-00014 12411795

[32] Vandenbussche E Duranthon LD Couturier M Pidhorz L Augereau B. The effect of tourniquet use in total knee arthroplasty [Clinical Trial; Journal Article; Randomized Controlled Trial]. Int Orthop. (2002) 26:306–9. 10.1007/s00264-002-0360-6 12378360 PMC3621001

[33] Wauke K Nagashima M Kato N Ogawa R Yoshino S. Comparative study between thromboembolism and total knee arthroplasty with or without tourniquet in rheumatoid arthritis patients. Arch Orthop Trauma Surg. (2002) 122:442–6. 10.1007/s00402-002-0404-9 12442180

[34] Katsumata S Nagashima M Kato K Tachihara A Wauke K Saito S . Changes in coagulation-fibrinolysis marker and neutrophil elastase following the use of tourniquet during total knee arthroplasty and the influence of neutrophil elastase on thromboembolism. Acta Anaesthesiol Scand. (2005) 49:510–6. 10.1111/j.1399-6576.2005.00621.x 15777299

[35] Matziolis G Drahn T Schröder JH Krocker D Tuischer J Perka C. Endothelin-1 is secreted after total knee arthroplasty regardless of the use of a tourniquet. J Orthop Res. (2005) 23:392–6. 10.1016/j.orthres.2004.08.021 15734253

[36] Nishiguchi M Takamura N Abe Y Kono M Shindo H Aoyagi K. Pilot study on the use of tourniquet: a risk factor for pulmonary thromboembolism after total knee arthroplasty? Thromb Res. (2005) 115:271–6. 10.1016/j.thromres.2004.08.018 15668186

[37] Fukuda A Hasegawa M Kato K Shi D Sudo A Uchida A. Effect of tourniquet application on deep vein thrombosis after total knee arthroplasty. Arch Orthop Trauma Surg. (2007) 127:671–5. 10.1007/s00402-006-0244-0 17102960

[38] Kageyama K Nakajima Y Shibasaki M Hashimoto S Mizobe T. Increased platelet, leukocyte, and endothelial cell activity are associated with increased coagulability in patients after total knee arthroplasty. J Thromb Haemost. (2007) 5:738–45. 10.1111/j.1538-7836.2007.02443.x 17408407

[39] Li B Qian QR Wu HS Zhao H Lin XB Zhu J . The use of a pneumatic tourniquet in total knee arthroplasty: a prospective, randomized study [English Abstract; Journal Article; Randomized Controlled Trial]. Zhonghua Wai Ke Za Zhi. (2008) 46:1054–7. 10.3321/j.issn:0529-5815.2008.14.005 19094529

[40] Yavarikia A Amjad GG Davoudpour K. The influence of tourniquet use and timing of its release on blood loss in total knee Arthroplasty. Pak J Biol Sci. (2010) 13:249–52. 10.3923/pjbs.2010.249.252 20464949

[41] Zhang FJ Xiao Y Liu YB Tian X Gao ZG. Clinical effects of applying a tourniquet in total knee arthroplasty on blood loss [Journal Article; Randomized Controlled Trial]. Chin Med J. (2010) 123:3030–3. 10.3760/cma.j.issn.0366-6999.2010.21.015 21162951

[42] Ledin H Aspenberg P Good L. Tourniquet use in total knee replacement does not improve fixation, but appears to reduce final range of motion [Journal Article; Randomized Controlled Trial; Research Support, Non-U.S. Gov't]. Acta Orthop. (2012) 83:499–503. 10.3109/17453674.2012.727078 22974220 PMC3488177

[43] Tai TW Chang CW Lai KA Lin CJ Yang CY. Effects of tourniquet use on blood loss and soft-tissue damage in total knee arthroplasty: a randomized controlled trial [Journal Article; Randomized Controlled Trial]. J Bone Joint Surg Am Vol. (2012) 94:2209–15. 10.2106/JBJS.K.00813 23318610

[44] Ejaz A Laursen AC Kappel A Laursen MB Jakobsen T Rasmussen S . Faster recovery without the use of a tourniquet in total knee arthroplasty [Journal Article; Randomized Controlled Trial]. Acta Orthop. (2014) 85:422–6. 10.3109/17453674.2014.931197 24954487 PMC4105775

[45] Liu D Graham D Gillies K Gillies RM. Effects of tourniquet use on quadriceps function and pain in total knee arthroplasty. Knee Surg Relat Res. (2014) 26:207–13. 10.5792/ksrr.2014.26.4.207 25505702 PMC4258487

[46] Dennis DA Kittelson AJ Yang CC Miner TM Kim RH Stevens-Lapsley JE. Does Tourniquet use in TKA affect recovery of lower extremity strength and function? A randomized trial [Journal Article; Randomized Controlled Trial]. Clin Orthop Relat Res. (2016) 474:69–77. 10.1007/s11999-015-4393-8 26100254 PMC4686529

[47] Liu PL Li DQ Zhang YK Lu QS Ma L Bao XZ . Effects of Unilateral Tourniquet used in patients undergoing simultaneous bilateral total knee arthroplasty [Journal Article; Randomized Controlled Trial]. Orthop Surg. (2017) 9:180–5. 10.1111/os.12329 28598560 PMC6584465

[48] Zhou K Ling T Wang H Zhou Z Shen B Yang J . Influence of tourniquet use in primary total knee arthroplasty with drainage: a prospective randomised controlled trial [Journal Article; Randomized Controlled Trial]. J Orthop Surg Res. (2017) 12:172. 10.1186/s13018-017-0683-z 29137681 PMC5686948

[49] Alexandersson M Wang EY Eriksson S. A small difference in recovery between total knee arthroplasty with and without tourniquet use the first 3 months after surgery: a randomized controlled study [Journal Article; Randomized Controlled Trial]. Knee Surg Sports Traumatol Arthrosc. (2018) 27:1035–42. 10.1007/s00167-018-5196-8 30328495 PMC6435610

[50] Ozkunt O Sariyilmaz K Gemalmaz HC Dikici F. The effect of tourniquet usage on cement penetration in total knee arthroplasty: a prospective randomized study of 3 methods. Medicine. (2018) 97:e9668. 10.1097/MD.0000000000009668 29369184 PMC5794368

[51] Jawhar A Skeirek D Stetzelberger V Kollowa K Obertacke U. No effect of tourniquet in primary total knee arthroplasty on muscle strength, functional outcome, patient satisfaction and health status: a randomized clinical trial [Journal Article; Randomized Controlled Trial]. Knee Surg Sports Traumatol Arthrosc. (2020) 28:1045–54. 10.1007/s00167-019-05646-5 31372679

[52] Zhao HY Yeersheng R Kang XW Xia YY Kang PD Wang WJ. The effect of tourniquet uses on total blood loss, early function, and pain after primary total knee arthroplasty a prospective, randomized controlled trial [Article]. Bone Joint Res. (2020) 9:322–32. 10.1302/2046-3758.96.BJR-2019-0180.R3 32670565 PMC7342055

[53] Danoff JR Goel R Sutton R Maltenfort MG Austin MS. How much pain is significant? Defining the minimal clinically important difference for the visual analog scale for pain after total joint arthroplasty. J Arthroplasty. (2018) 33:S71–5.e2. 10.1016/j.arth.2018.02.029 29567002

[54] Guler O Mahirogullari M Isyar M Piskin A Yalcin S Mutlu S Sahin B. Comparison of quadriceps muscle volume after unilateral total knee arthroplasty with and without tourniquet use. Knee Surg Sports Traumatol Arthrosc. (2016) 24:2595–605. 10.1007/s00167-015-3872-5 26590567

[55] Worland RL Arredondo J Angles F Lopez-Jimenez F Jessup DE. Thigh pain following tourniquet application in simultaneous bilateral total knee replacement arthroplasty. J Arthroplasty. (1997) 12:848–52. 10.1016/S0883-5403(97)90153-4 9458249

[56] Ajnin S Fernandes R. Reduced length of stay and faster recovery after total knee arthroplasty without the use of tourniquet. J Clin Orthop Trauma. (2020) 11:129–32. 10.1016/j.jcot.2019.08.016 32002000 PMC6985009

[57] Nielsen CS Jans Ø Ørsnes T Foss NB Troelsen A Husted H. Combined intra-articular and intravenous tranexamic acid reduces blood loss in total knee arthroplasty: a randomized, double-blind, placebo-controlled trial. J Bone Joint Surg Am Vol. (2016) 98:835–41. 10.2106/JBJS.15.00810 27194493

[58] Schnettler T Papillon N Rees H. Use of a Tourniquet in total knee arthroplasty causes a paradoxical increase in total blood loss. J Bone Joint Surg Am Vol. (2017) 99:1331–6. 10.2106/JBJS.16.00750 28816892

[59] Rama KR Apsingi S Poovali S Jetti A. Timing of tourniquet release in knee arthroplasty. Meta-analysis of randomized, controlled trials. J Bone Joint Surg Am Vol. (2007) 89:699–705. 10.2106/00004623-200704000-00001 17403789

[60] Mori N Kimura S Onodera T Iwasaki N Nakagawa I Masuda T. Use of a pneumatic tourniquet in total knee arthroplasty increases the risk of distal deep vein thrombosis: a prospective, randomized study. Knee. (2016) 23:887–9. 10.1016/j.knee.2016.02.007 27372555

[61] Liu Y Si H Zeng Y Li M Xie H Shen B. More pain and slower functional recovery when a tourniquet is used during total knee arthroplasty. Knee Surg Sports Traumatol Arthrosc. (2020) 28:1842–60. 10.1007/s00167-019-05617-w 31289914

[62] Ricciardi BF Oi KK Daines SB Lee YY Joseph AD Westrich GH. Patient and perioperative variables affecting 30-day readmission for surgical complications after hip and knee arthroplasties: a matched cohort study. J Arthroplasty. (2017) 32:1074–9. 10.1016/j.arth.2016.10.019 27876255

[63] Tie K Hu D Qi Y Wang H Chen L. Effects of Tourniquet release on total knee arthroplasty. Orthopedics. (2016) 39:e642–50. 10.3928/01477447-20160606-03 27286051

[64] Drolet M Bénard É Pérez N Brisson M. Population-level impact and herd effects following the introduction of human papillomavirus vaccination programmes: updated systematic review and meta-analysis. Lancet. (2019) 394:497–509. 10.1016/S0140-6736(19)30298-3 31255301 PMC7316527

[65] Packer M. Are meta-analyses a form of medical fake news? Thoughts about how they should contribute to medical science and practice. Circulation. (2017) 136:2097–9. 10.1161/CIRCULATIONAHA.117.030209 29180491

