# Supplementary material for: Association of Use of Tourniquets During Total Knee Arthroplasty in the Elderly Patients With Post-operative Pain and Return to Function
Source: Front Public Health. 2022 Mar 10;10:825408. doi: 10.3389/fpubh.2022.825408 (PMC8960992; doi:10.3389/fpubh.2022.825408)
Supplement: Supplementary file 1 [file Table_1.docx]

**Supplementary files**

**1. Figures** (Supplementary Figure 1- Supplementary Figure 23)

**Supplementary Figure 1.** Literature Search and Screening Process.

**Supplementary Figure 2.** Risk of bias summary of included studies.

**Supplementary Figure 3.** Risk of bias of included studies.

**Supplementary Figure 4.** Contour-enhanced funnel plot of ROM.

**Supplementary Figure 5.** Subgroup analysis of ROM on the third postoperative day.

**Supplementary Figure 6.** Subgroup analysis of ROM on the first postoperative month.

**Supplementary Figure 7.** Subgroup analysis of ROM on the third postoperative month.

**Supplementary Figure 8.** Contour-enhanced funnel plot of pain.

**Supplementary Figure 9.** Subgroup analysis of pain on third postoperative day.

**Supplementary Figure 10.** Subgroup analysis of pain on the first postoperative month.

**Supplementary Figure 11.** The forest plot regarding intraoperative blood loss and postoperative blood loss.

**Supplementary Figure 12.** Contour-enhanced funnel plot of intraoperative blood loss.

**Supplementary Figure 13.** Subgroup analysis of intraoperative blood loss.

**Supplementary Figure 14.** Contour-enhanced funnel plot of postoperative blood loss.

**Supplementary Figure 15.** Subgroup analysis of postoperative blood loss.

**Supplementary Figure 16.** Contour-enhanced funnel plot of measured total blood loss.

**Supplementary Figure 17.** Subgroup analysis of measured total blood loss.

**Supplementary Figure 18.** Contour-enhanced funnel plot of calculated total blood loss.

**Supplementary Figure 19.** Subgroup analysis of calculated total blood loss.

**Supplementary Figure 20.** The forest plot regarding operation time compared no tourniquet with tourniquet group.

**Supplementary Figure 21.** Contour-enhanced funnel plot of operation time.

**Supplementary Figure 22.** Subgroup analysis of operation time.

**Supplementary Figure 23.** Contour-enhanced funnel plot of transfusion rate.

**Supplementary Figure 24.** Contour-enhanced funnel plot of DVT.

**Supplementary Figure 25.** Contour-enhanced funnel plot of superfical wound infection rate.

**Supplementary Figure 26.** Contour-enhanced funnel plot of all complication rate.

**Supplementary Figure 27.** The forest plot regarding pulmonary embolism compared no tourniquet with tourniquet group.

**Supplementary Figure 28.** Contour-enhanced funnel plot of pulmonary embolism.

**2. Tables** (Supplementary Table 1- Supplementary Table 5)

**Supplementary Table 1.** Search strategy.

**Supplementary Table 2.** Inclusion/exclusion criteria of literature.

**Supplementary Table 3.** Risk of bias table.

**Supplementary Table 4.** Scores of the Newcastle-Ottawa Quality Assessment Scale for 16 cohort studies.

**Supplementary Table 5.** Characteristics of the Included Trials and Participants.

**Supplementary Figure 1. Literature Search and Screening Process.**


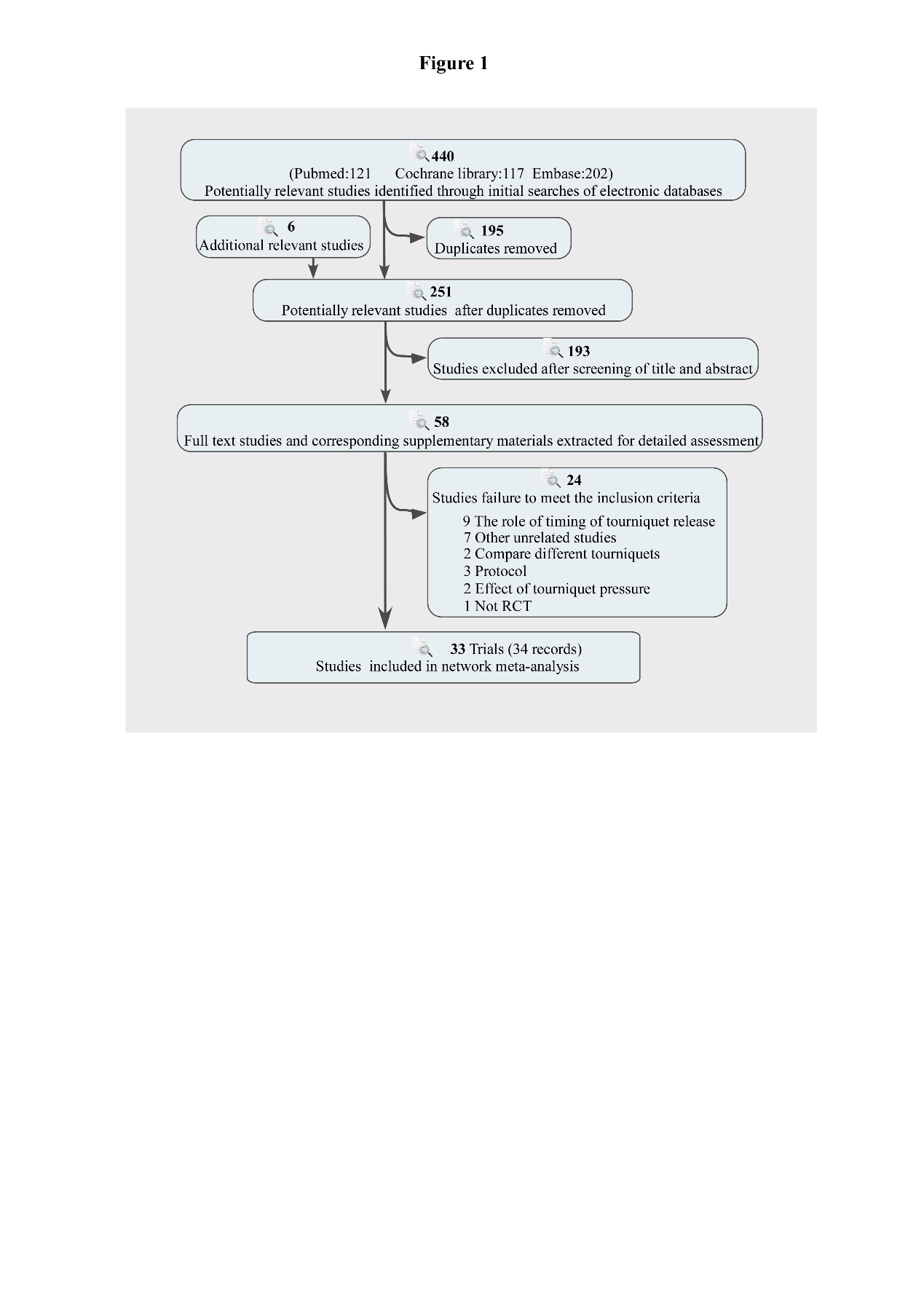


**Supplementary Figure 2. Risk of bias summary of included studies.**


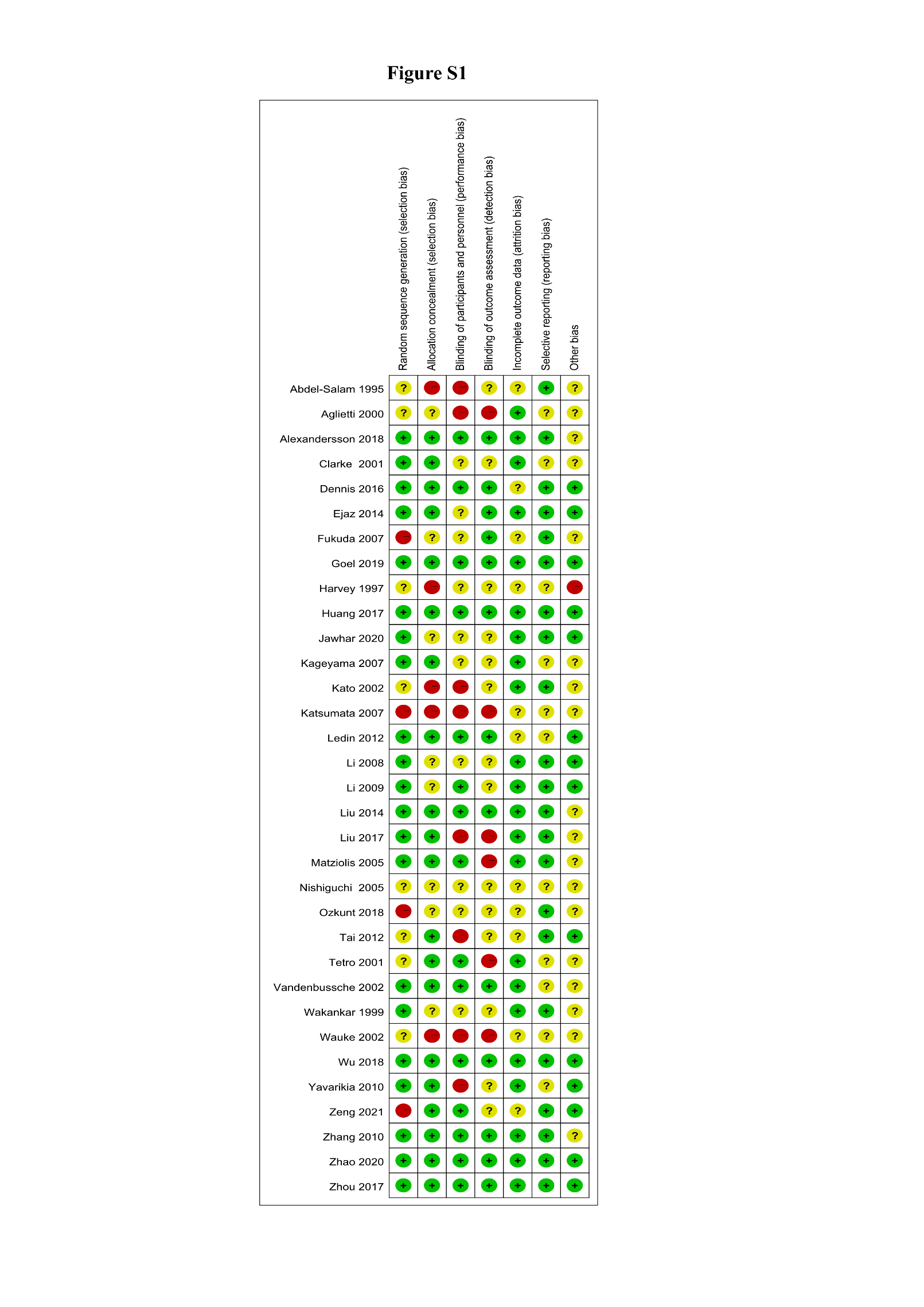


**Supplementary Figure 3.** **Risk of bias of included studies.**


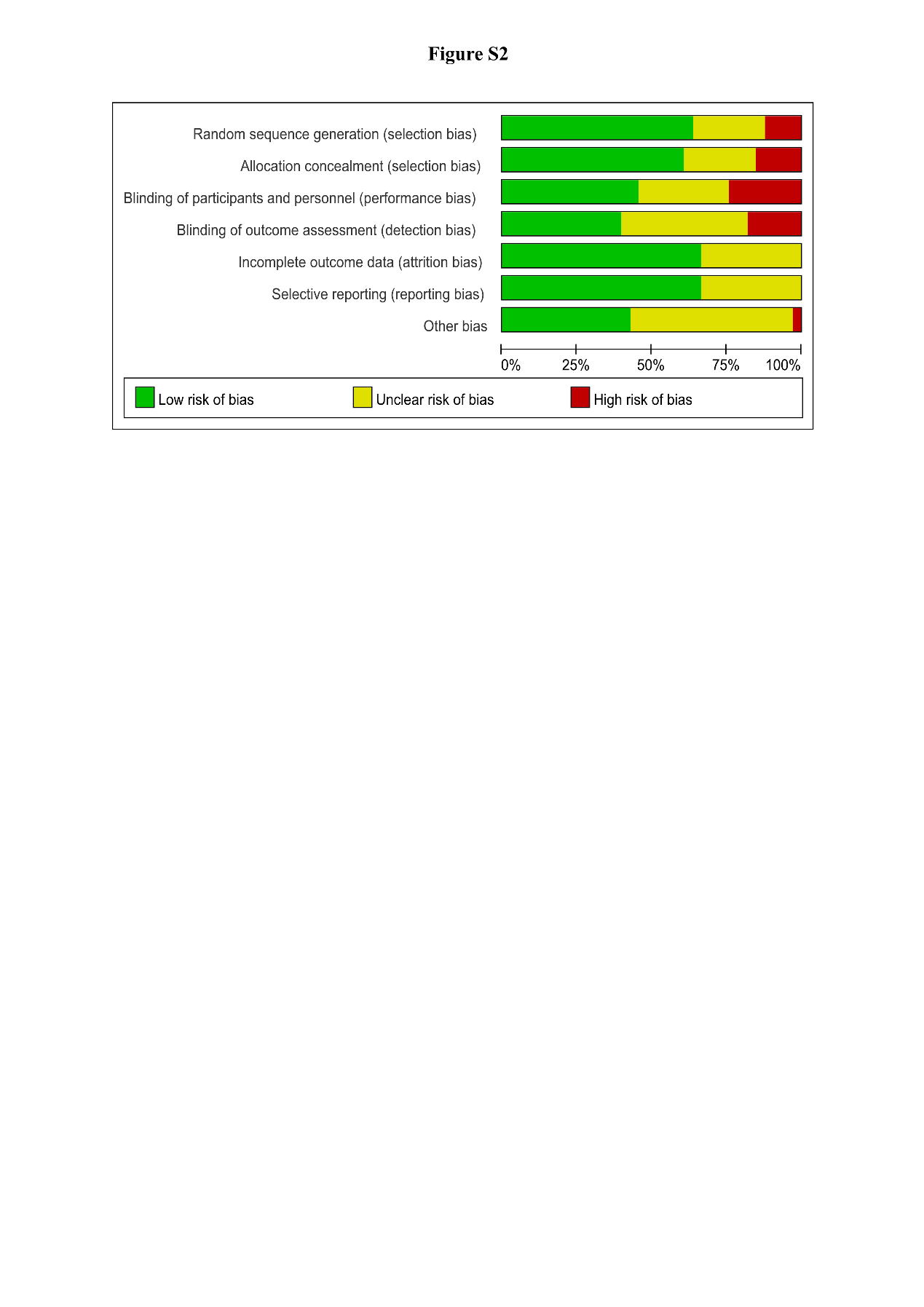


**Supplementary Figure 4.** **Contour-enhanced funnel plot of ROM.**


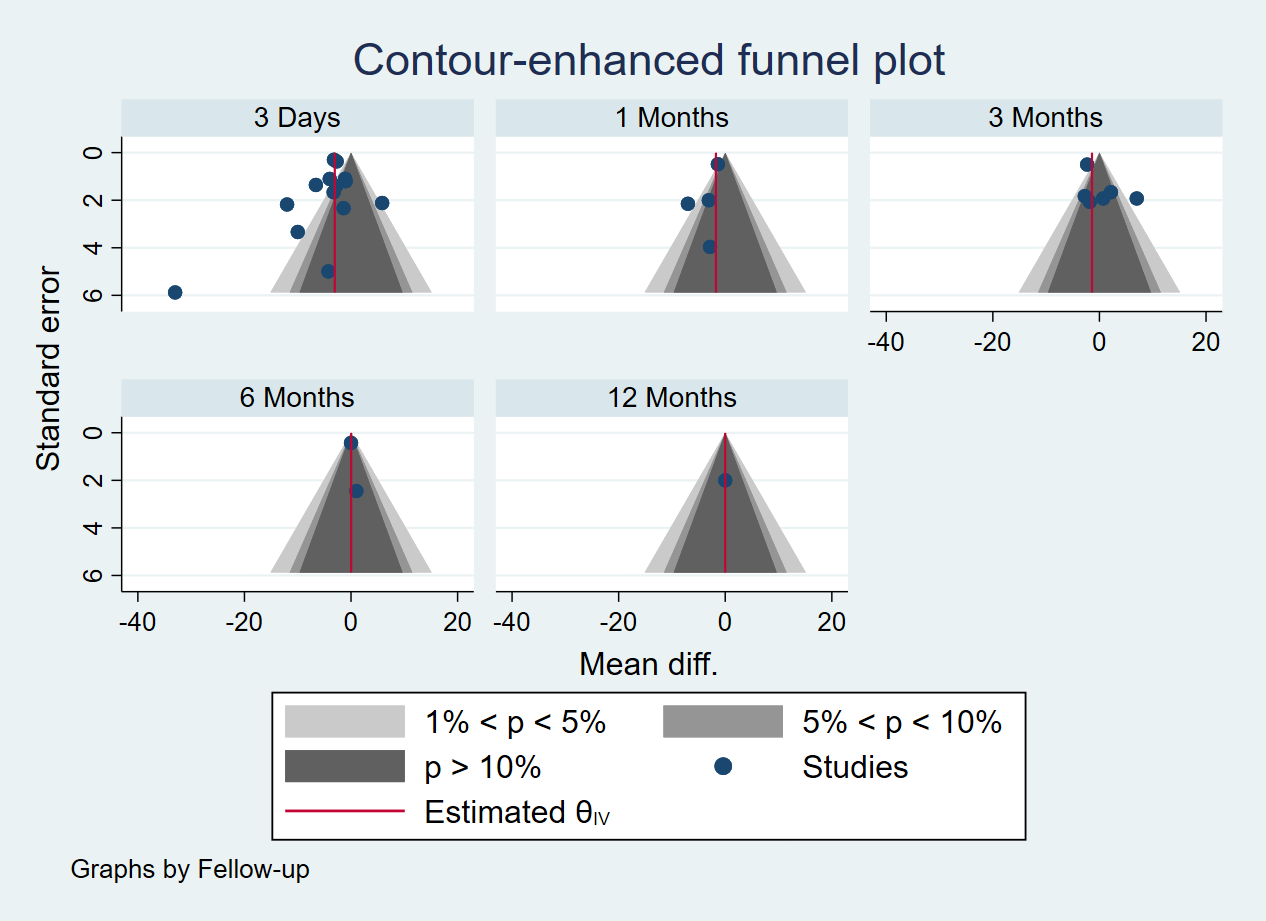


**Supplementary Figure 5. Subgroup analysis of ROM on the third postoperative day.**


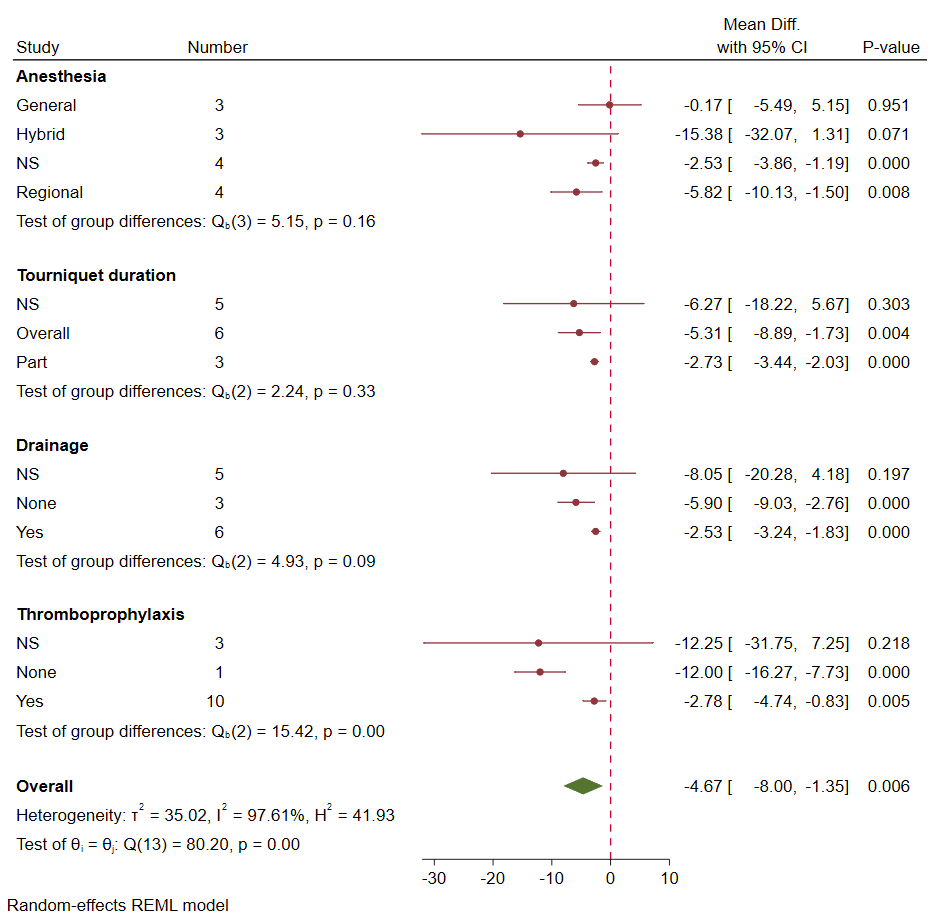


**Supplementary Figure 6.** **Subgroup analysis of ROM on the first postoperative month.**


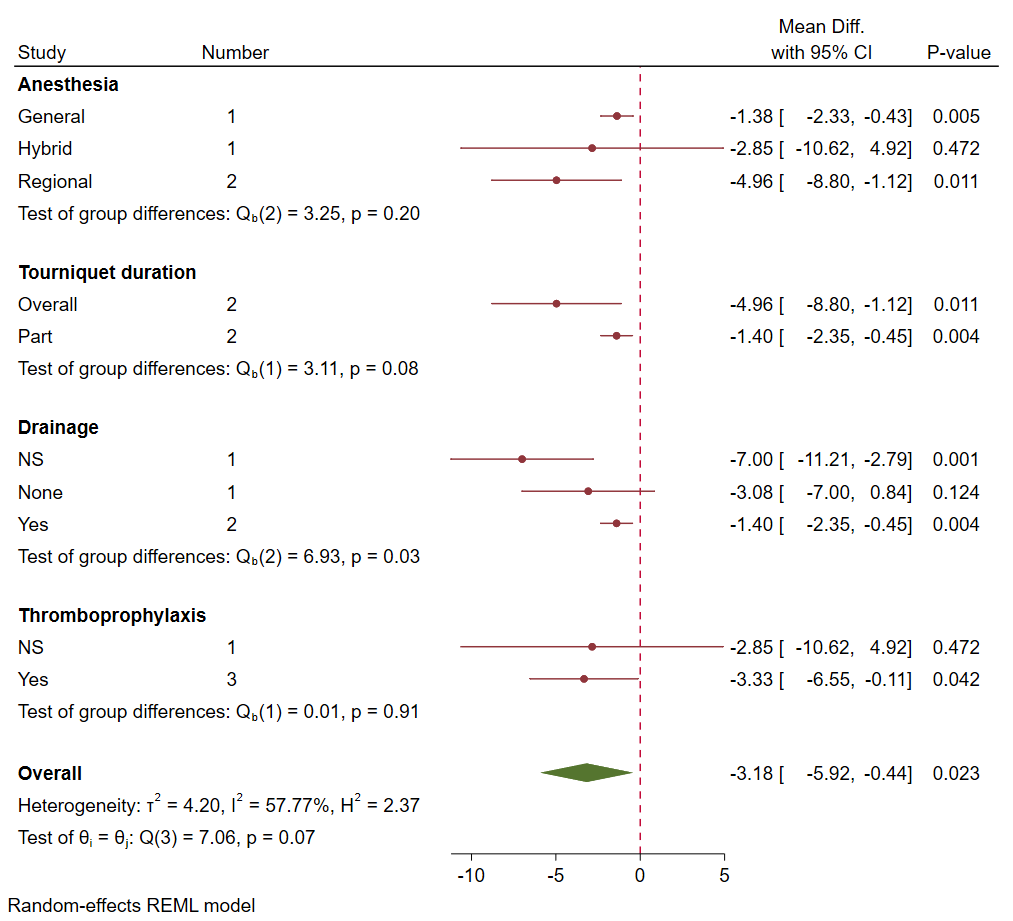


**Supplementary Figure 7.** **Subgroup analysis of ROM on the third postoperative month.**


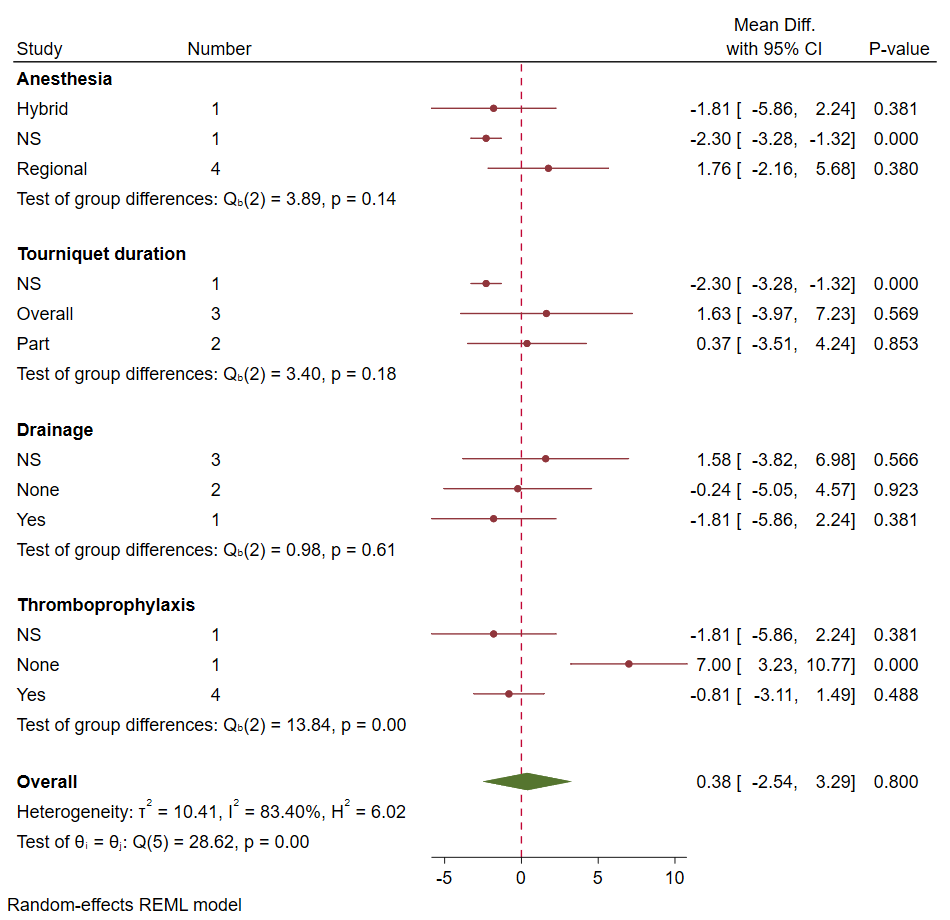


**Supplementary Figure 8.** **Contour-enhanced funnel plot of pain.**


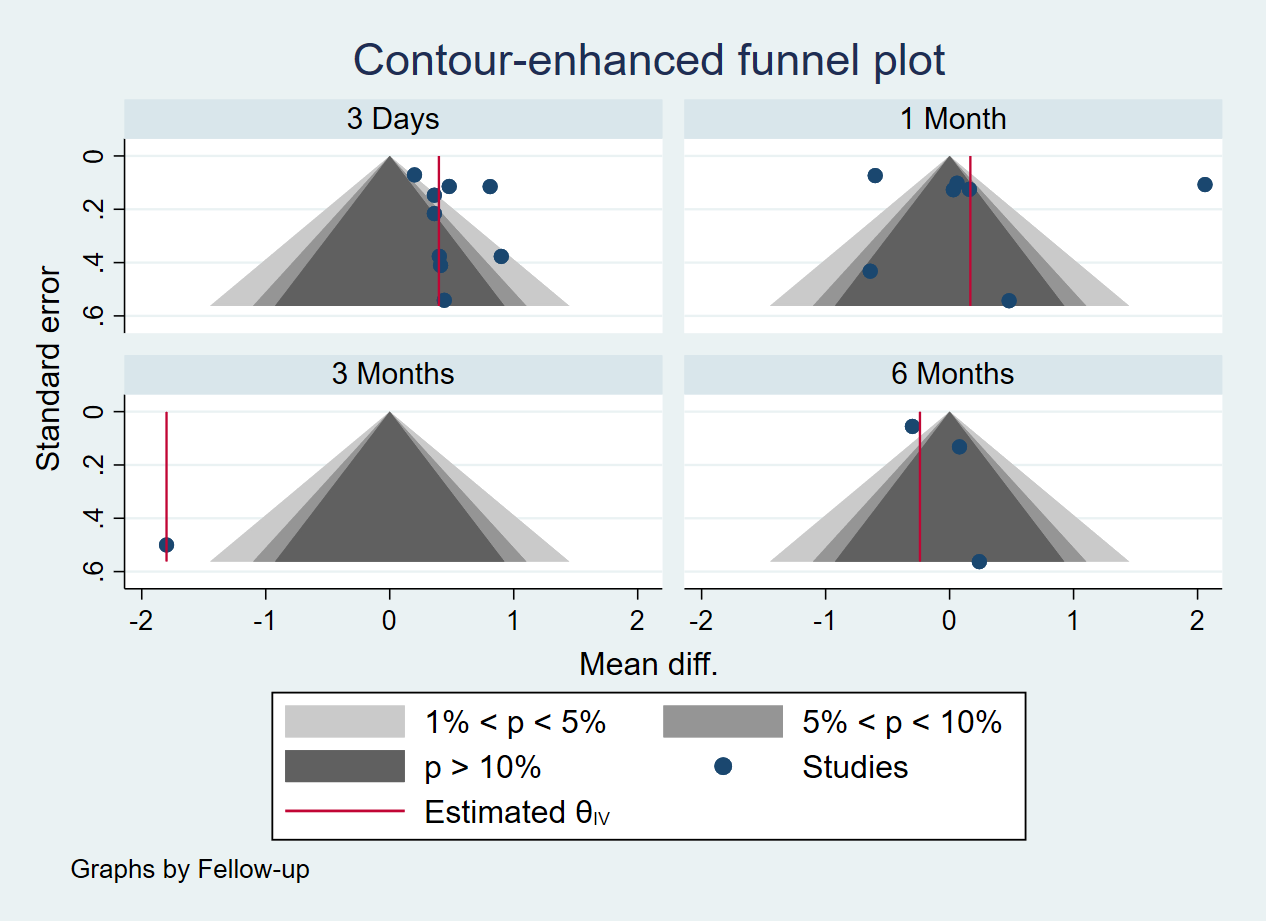


**Supplementary Figure 9.** **Subgroup analysis of pain on third postoperative day.**


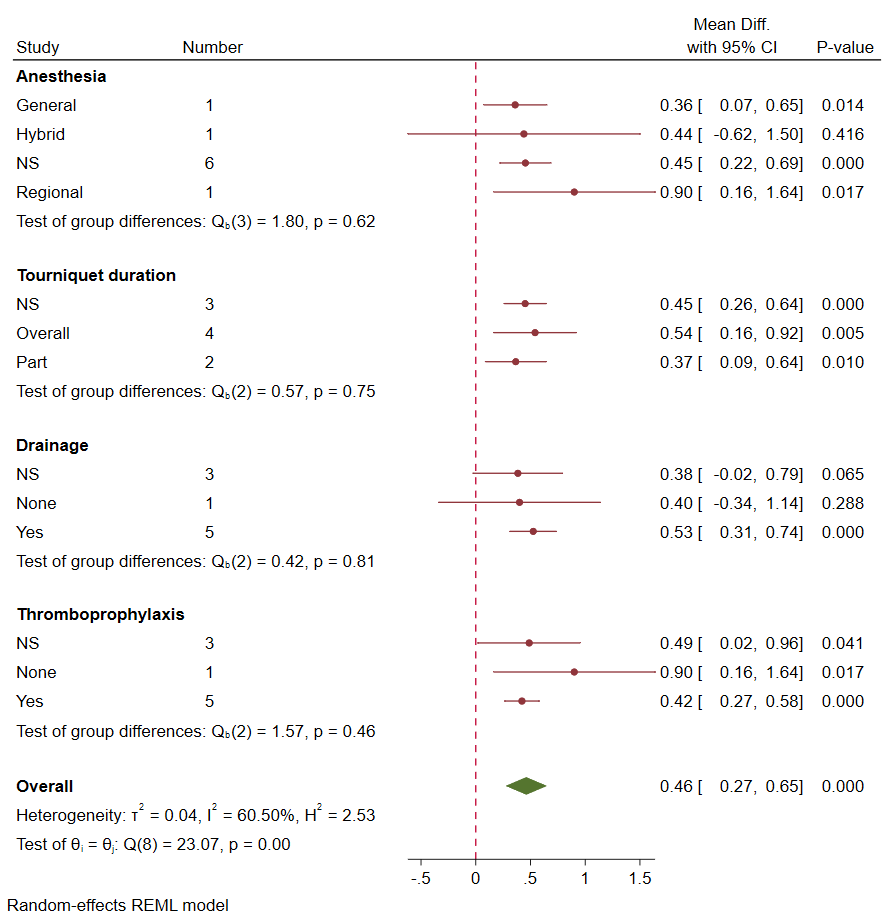


**Supplementary Figure 10.** **Subgroup analysis of pain on the first postoperative month.**


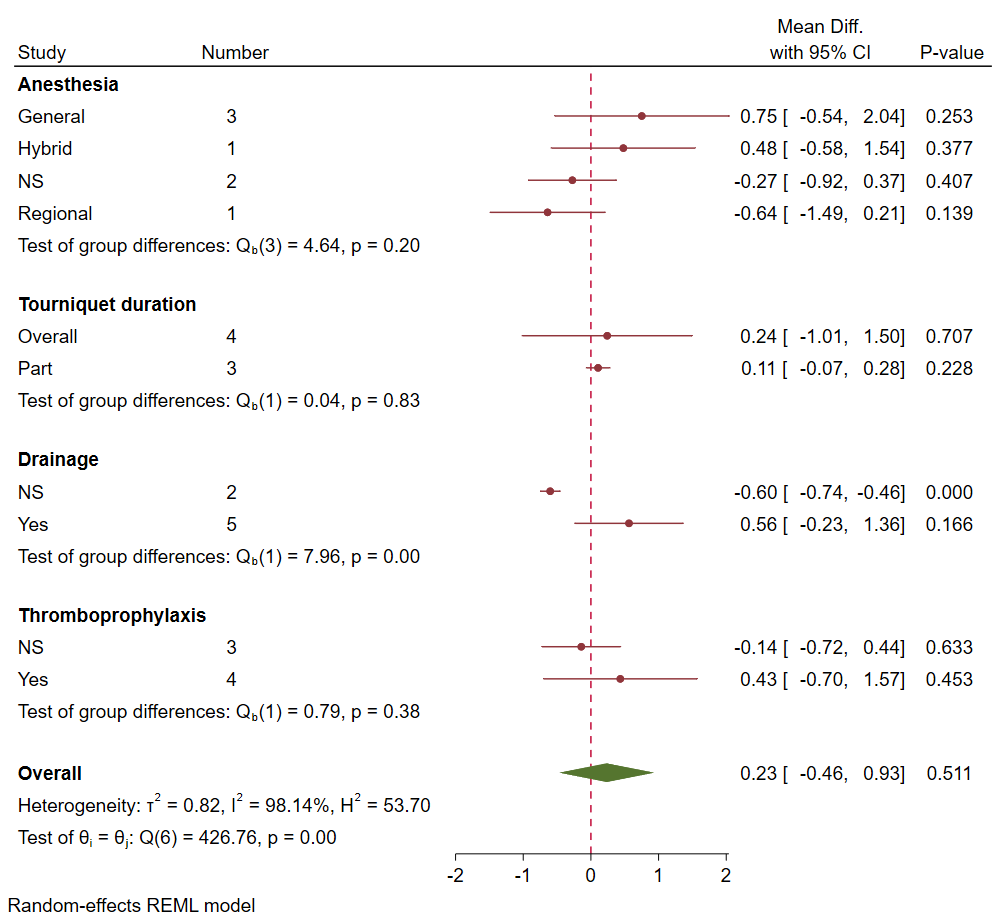


**Supplementary Figure 11. The forest plot regarding intraoperative blood loss and postoperative blood loss.**


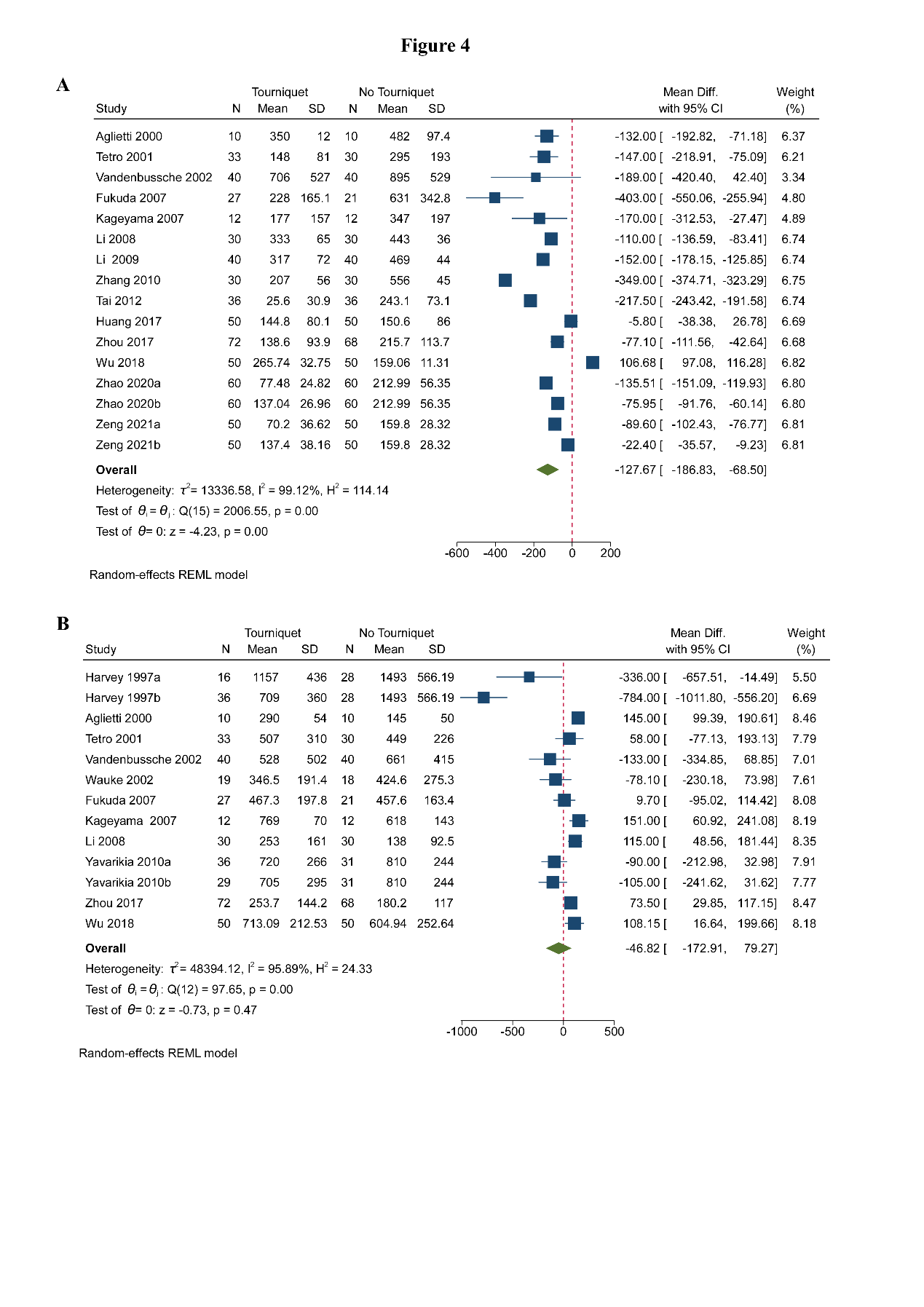


(A). The forest plot regarding intraoperative blood loss compared no tourniquet with tourniquet group; (B). The forest plot regarding postoperative blood loss compared no tourniquet with tourniquet group.

**Supplementary Figure 12.** **Contour-enhanced funnel plot of intraoperative blood loss.**


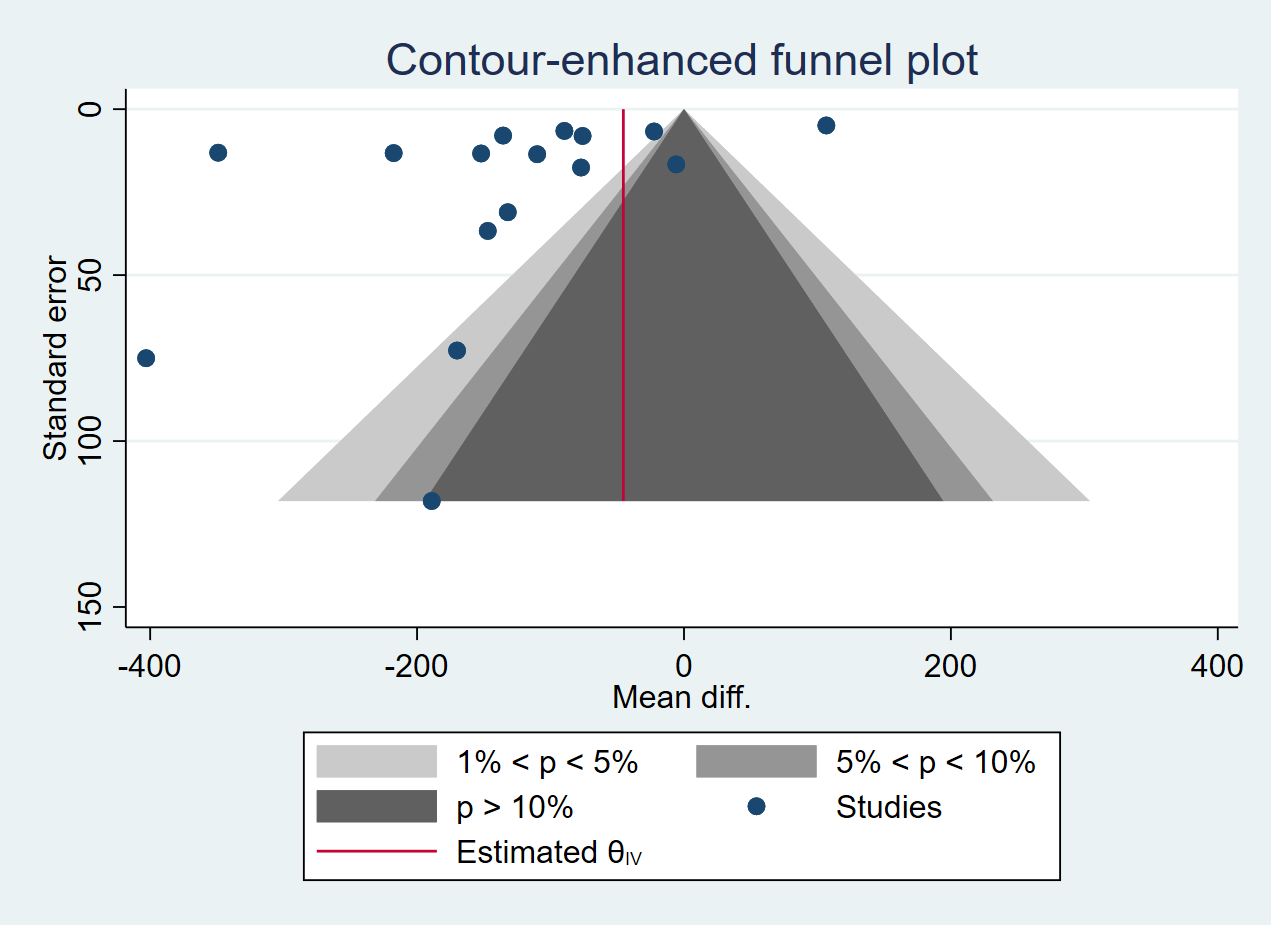


**Supplementary Figure 13.** **Subgroup analysis of intraoperative blood loss.**


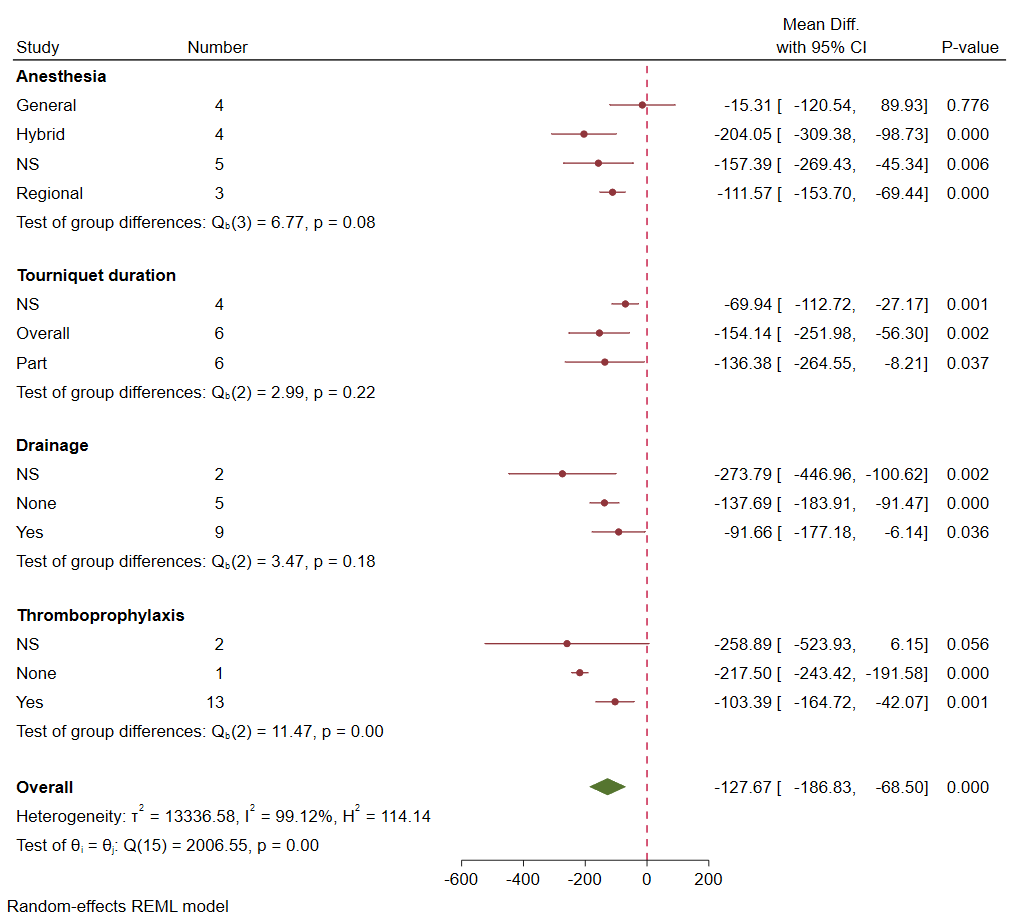


**Supplementary Figure 14.** **Contour-enhanced funnel plot of postoperative blood loss.**


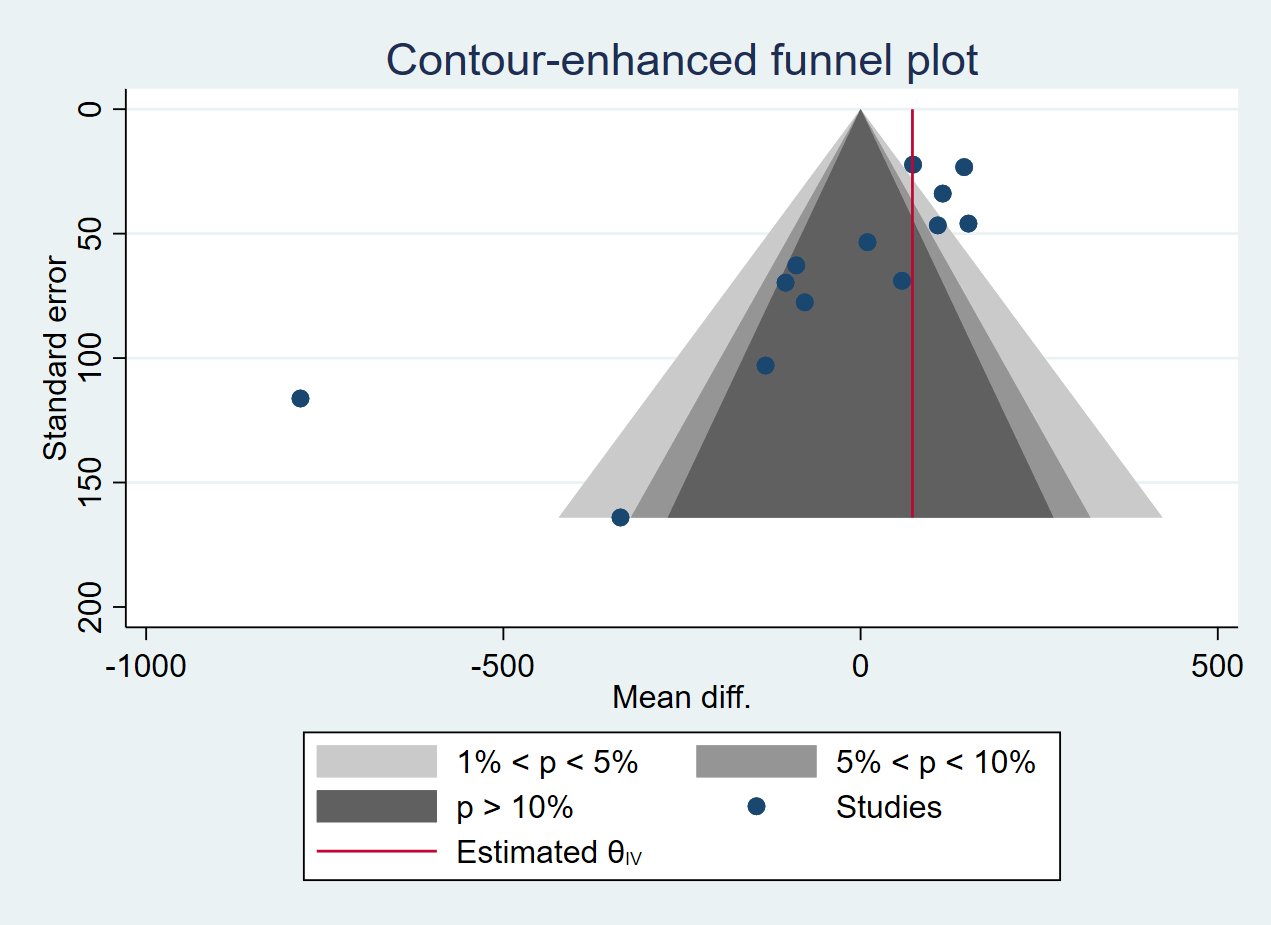


**Supplementary Figure 15.** **Subgroup analysis of postoperative blood loss.**


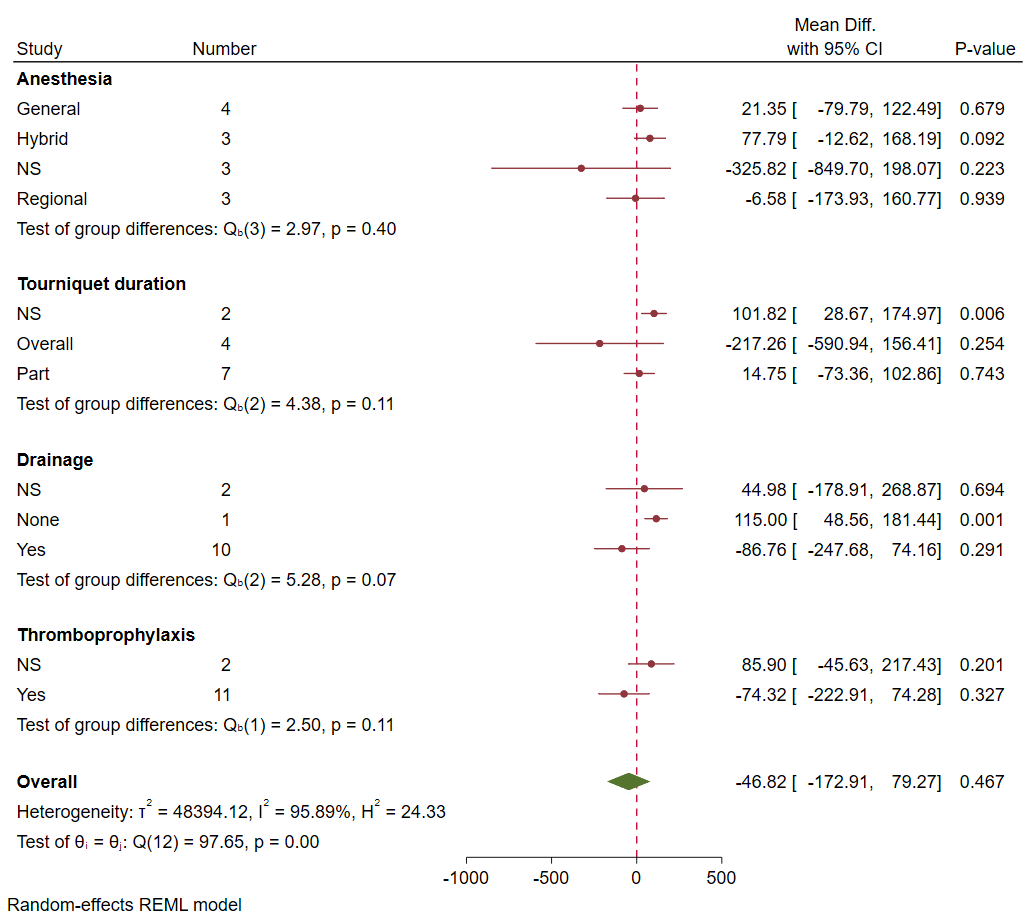


**Supplementary Figure 16.** **Contour-enhanced funnel plot of measured total blood loss.**


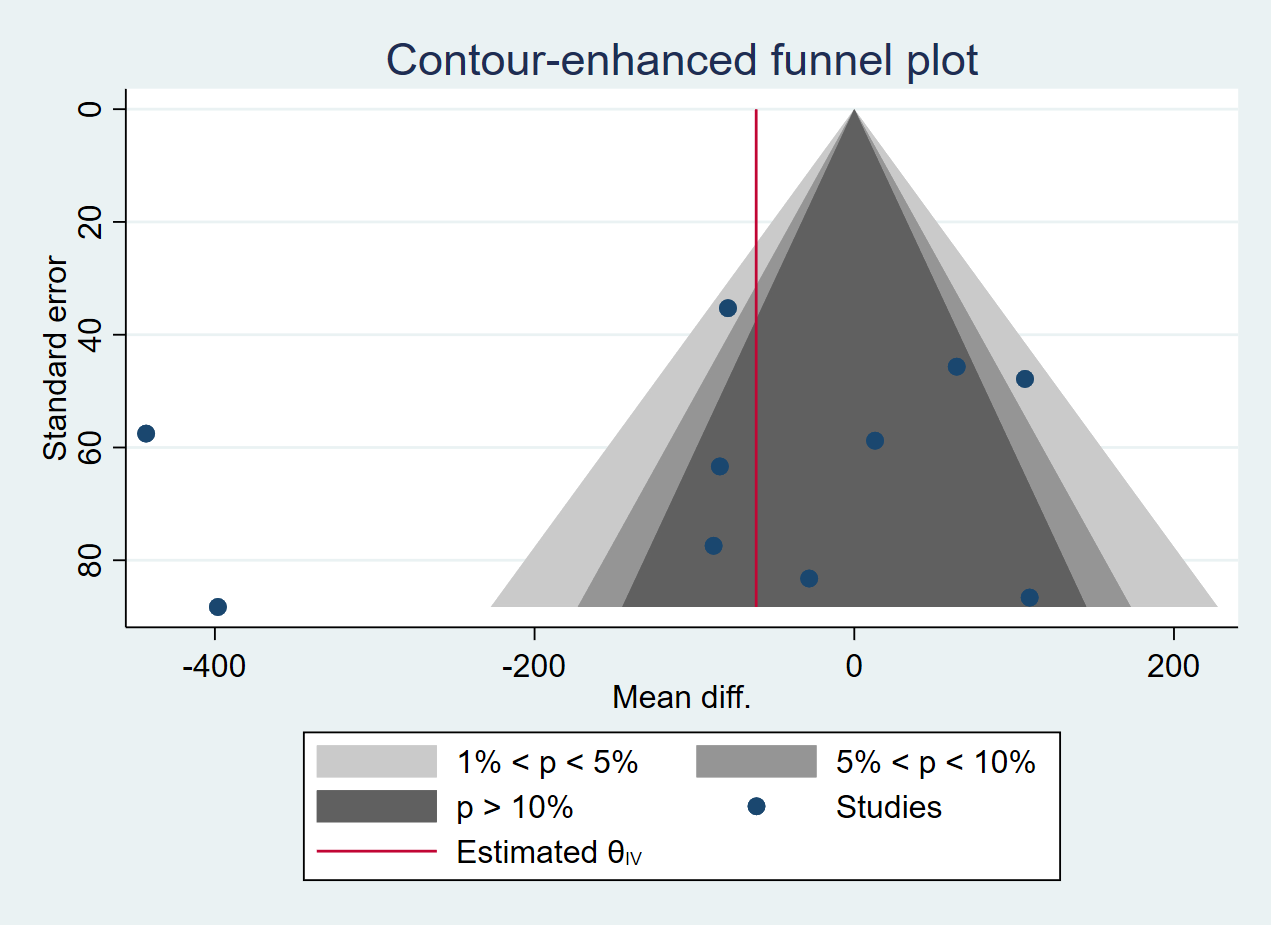


**Supplementary Figure 17.** **Subgroup analysis of measured total blood loss.**


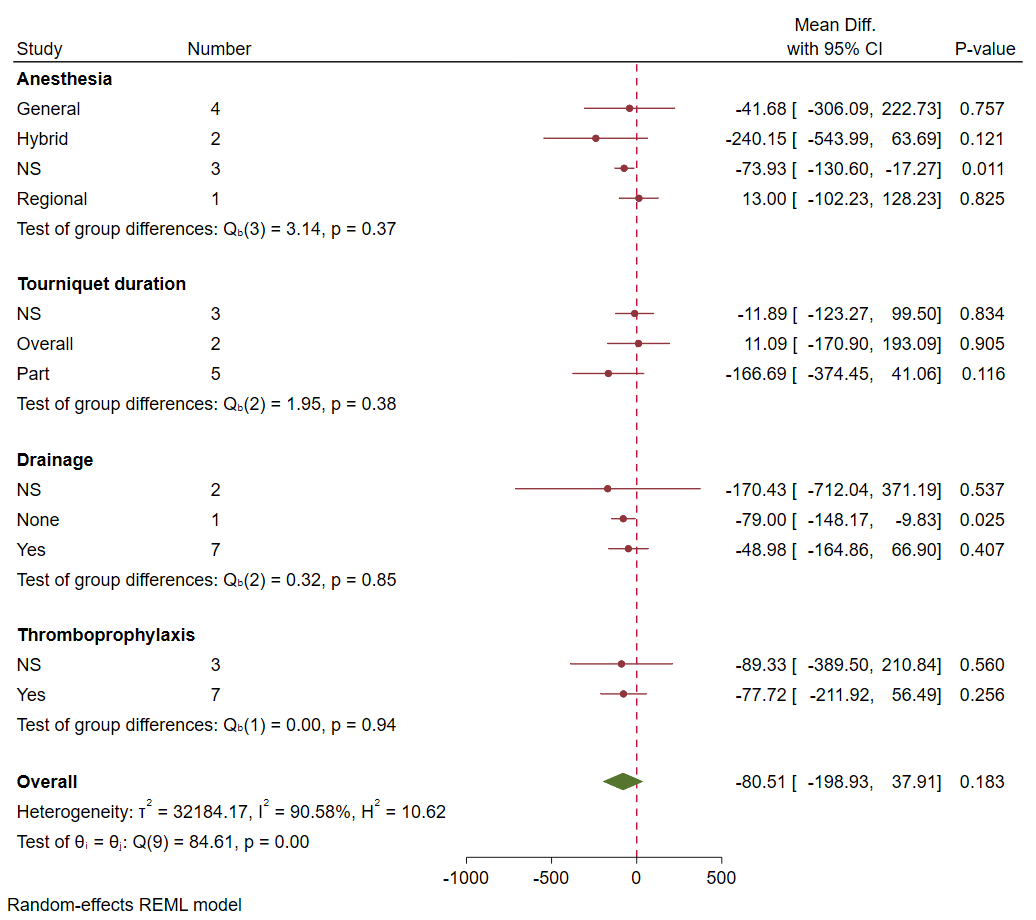


**Supplementary Figure 18.** **Contour-enhanced funnel plot of calculated total blood loss.**


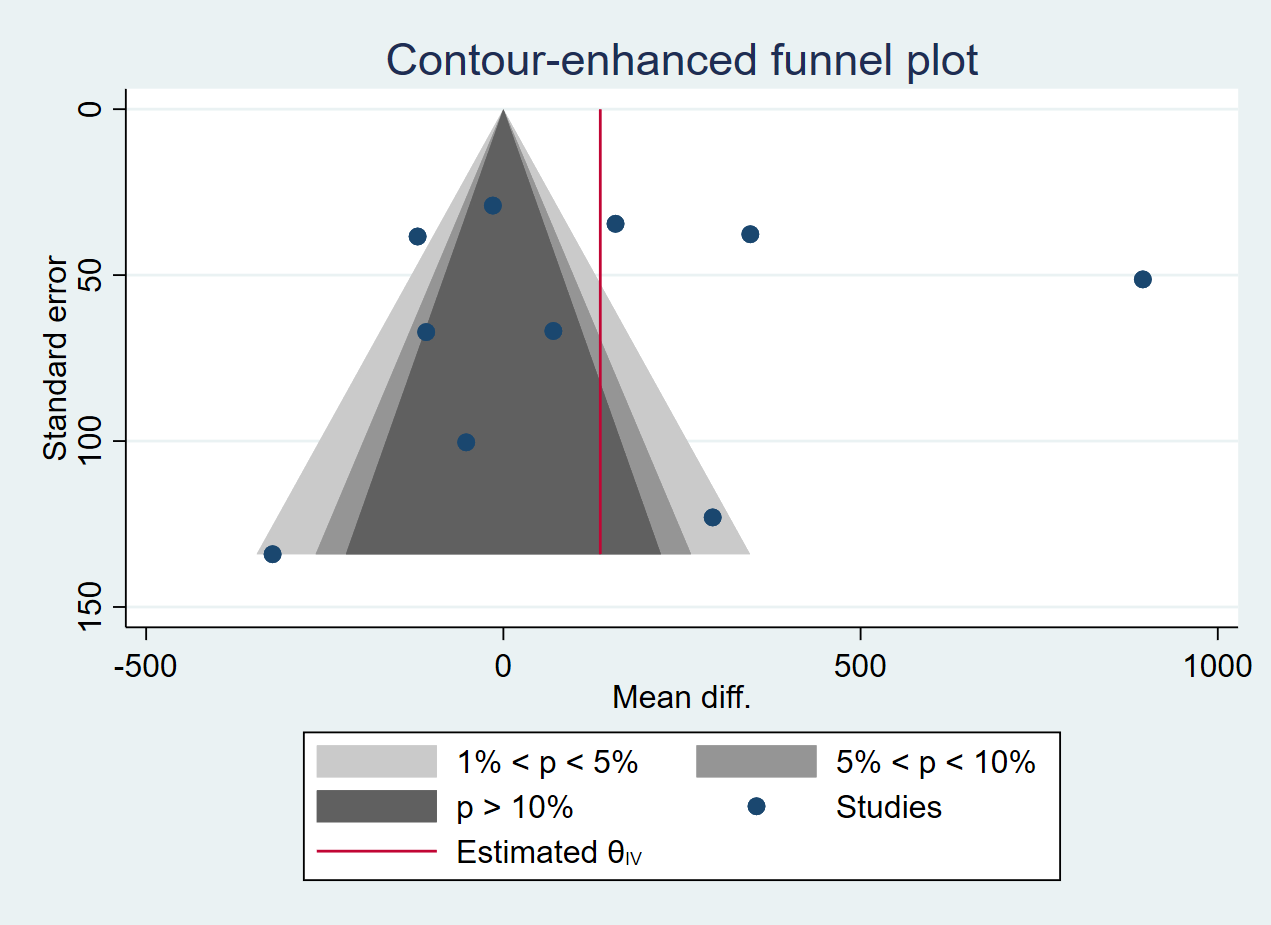


**Supplementary Figure 19. Subgroup analysis of calculated total blood loss.**


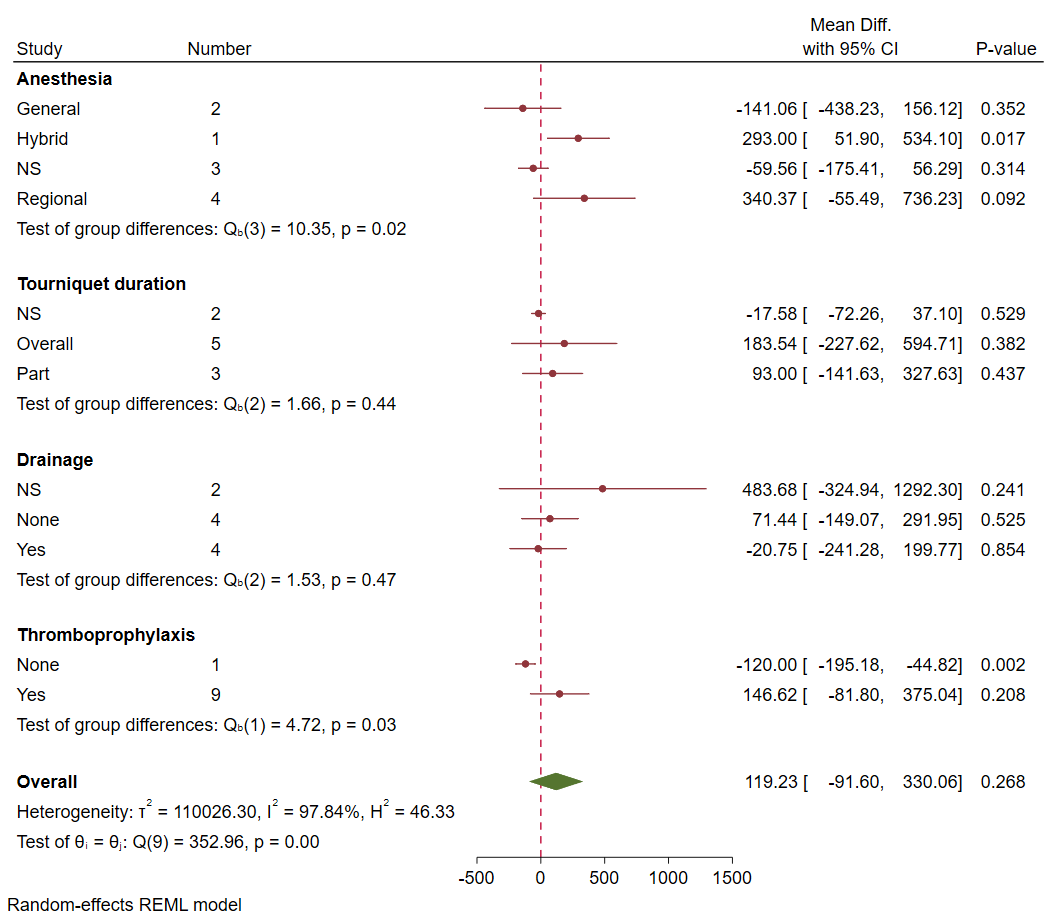


**Supplementary Figure 20. The forest plot regarding operation time compared no tourniquet with tourniquet group.**


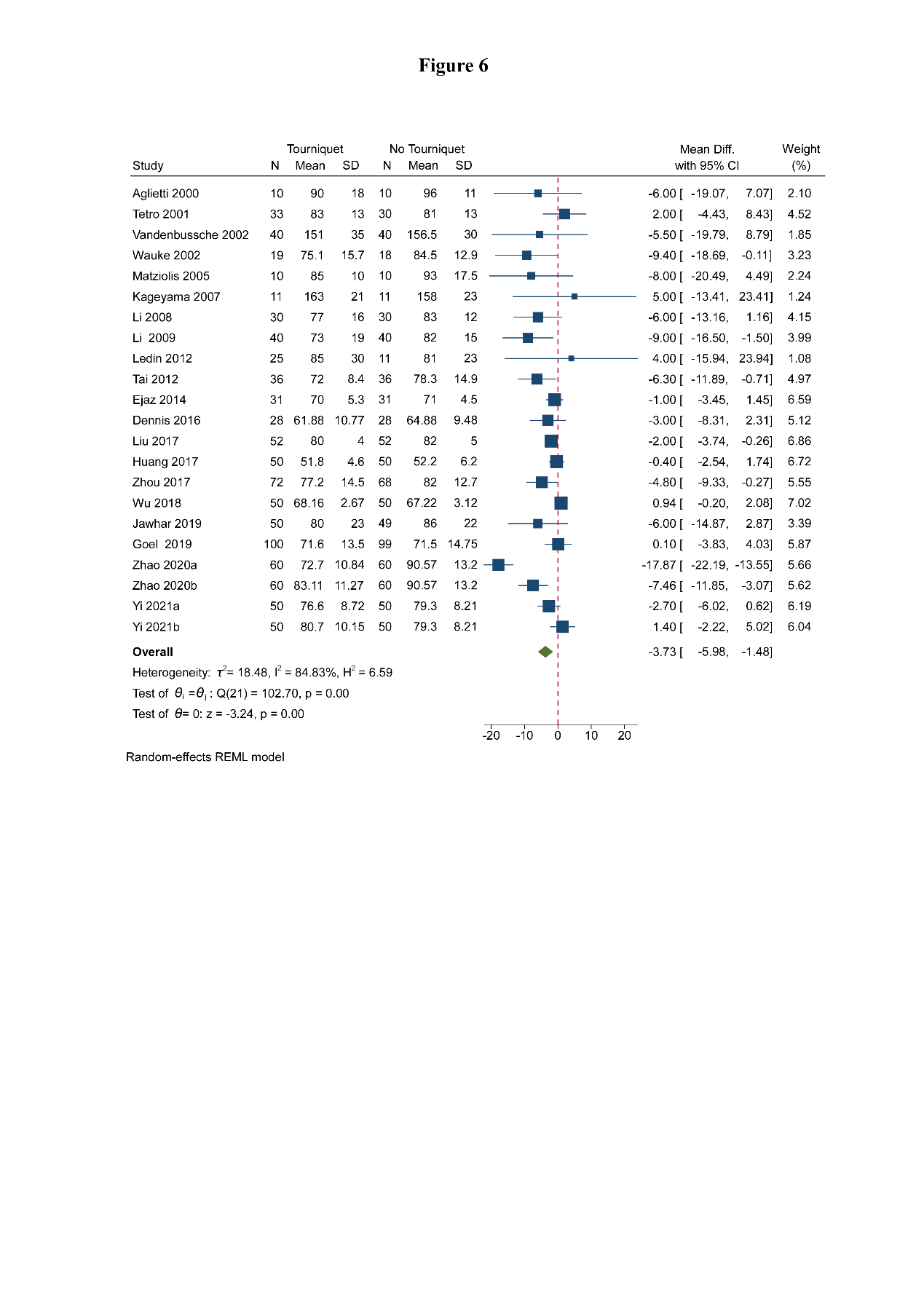


**Supplementary Figure 21. Contour-enhanced funnel plot of operation time.**


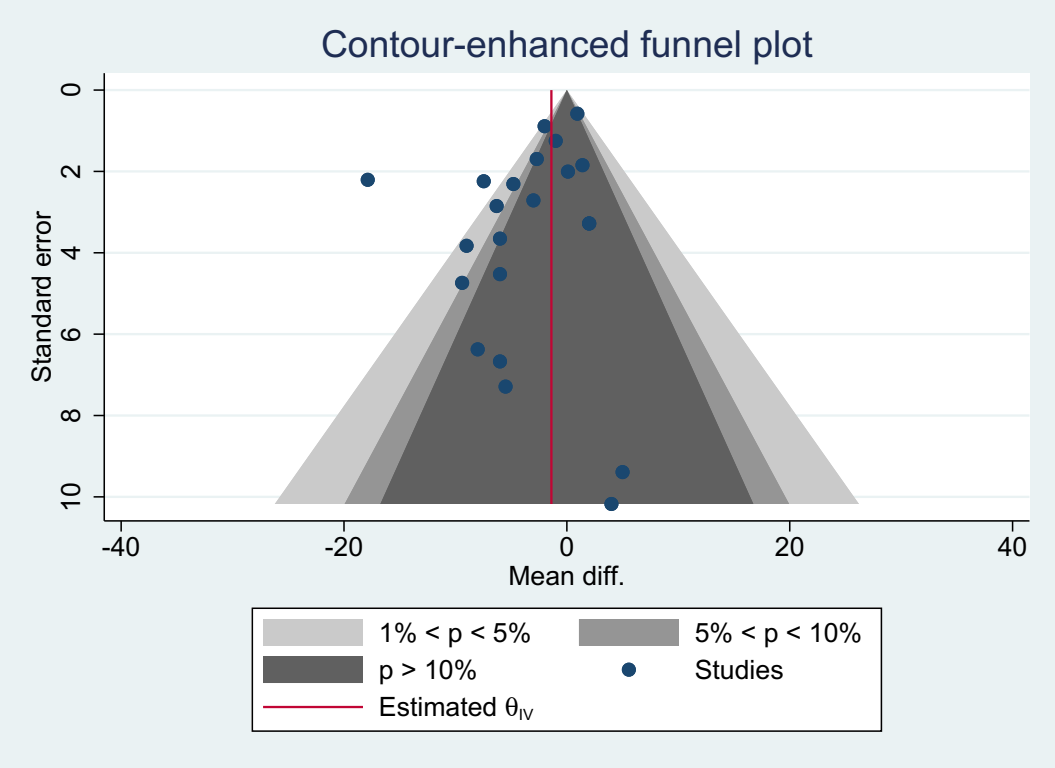


**Supplementary Figure 22.** **Subgroup analysis of** **operation time.**


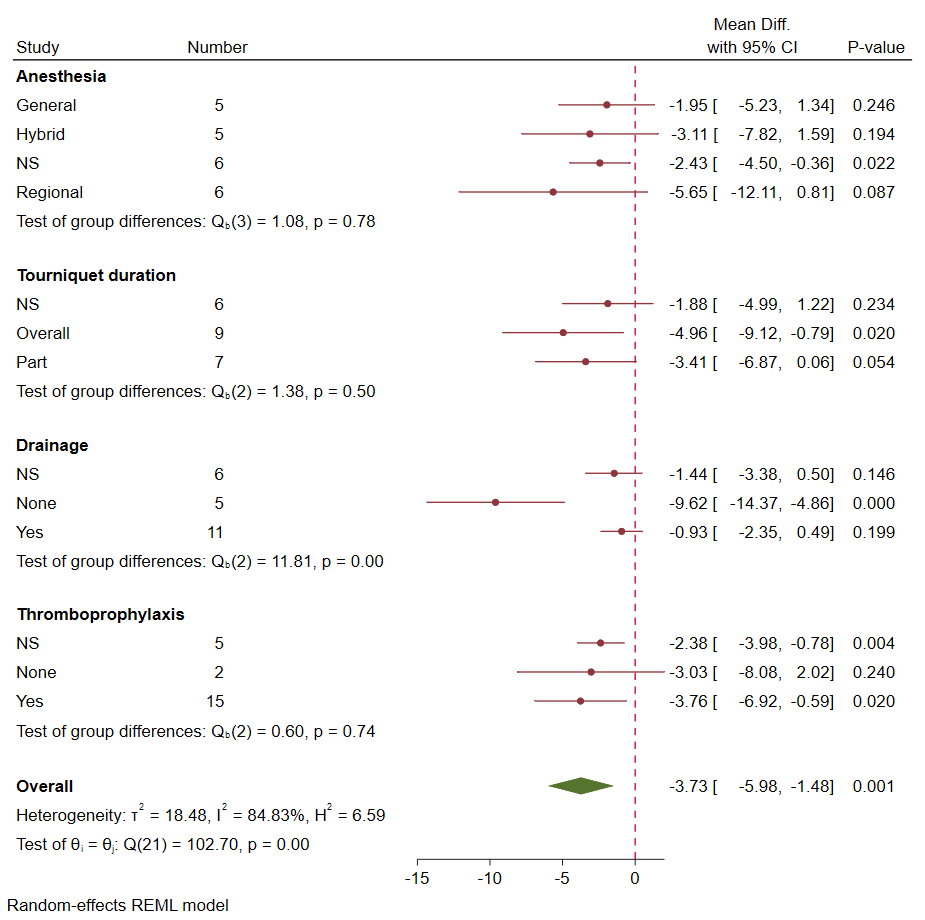


**Supplementary Figure 23. Contour-enhanced funnel plot of transfusion rate.**


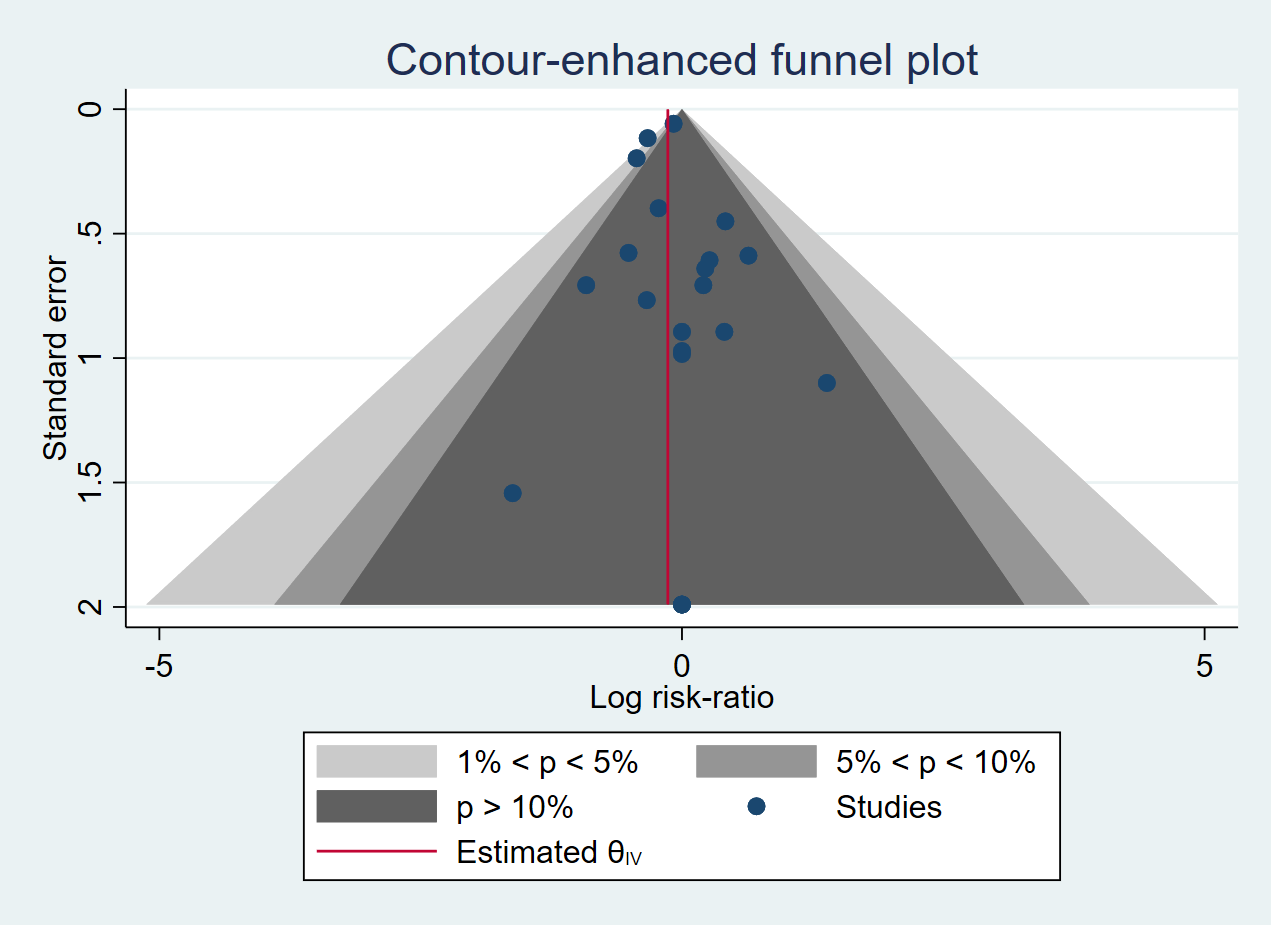


**Supplementary Figure 24.** **Contour-enhanced funnel plot of DVT.**


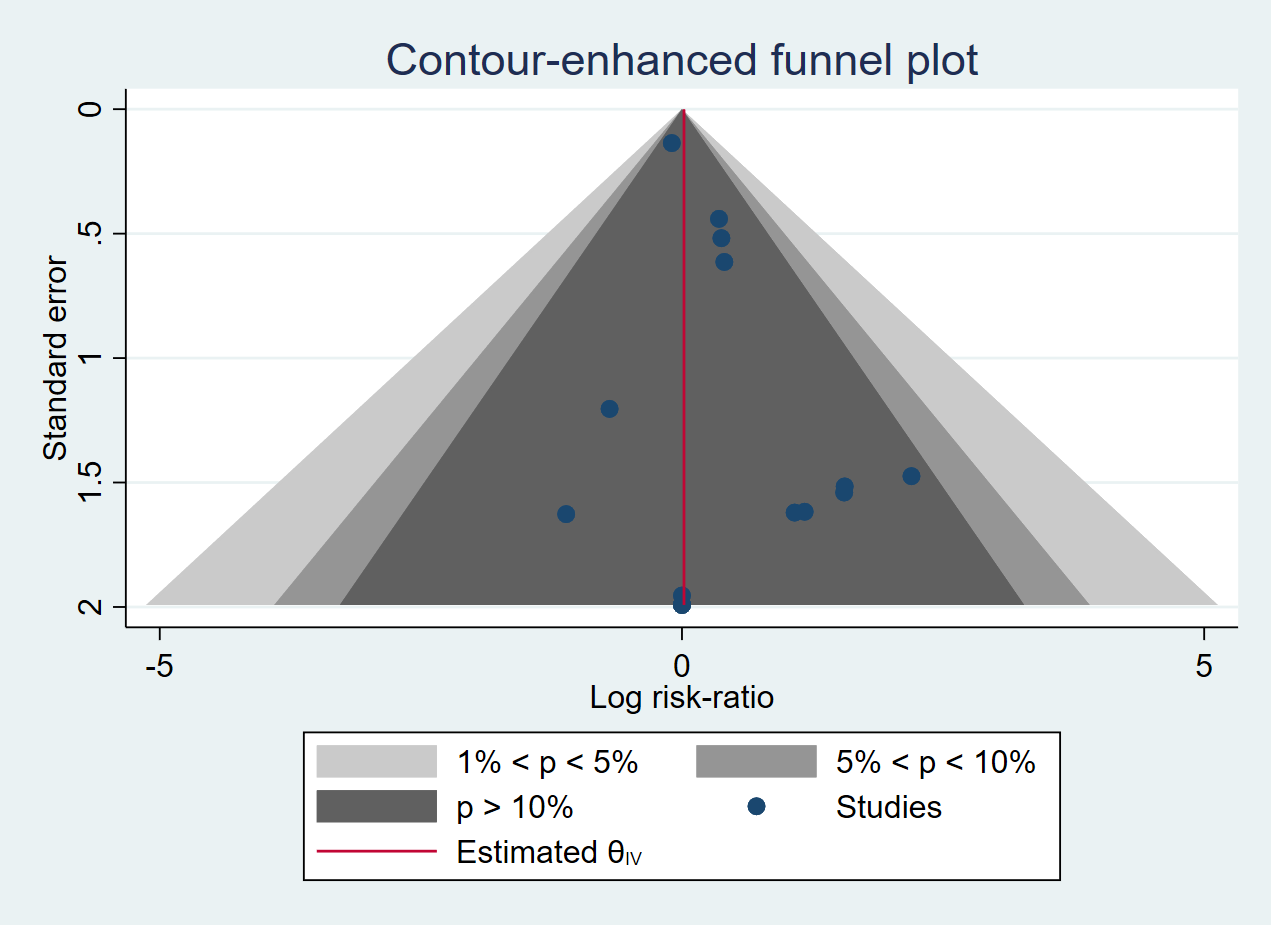


**Supplementary Figure 25. Contour-enhanced funnel plot of superfical wound infection rate.**


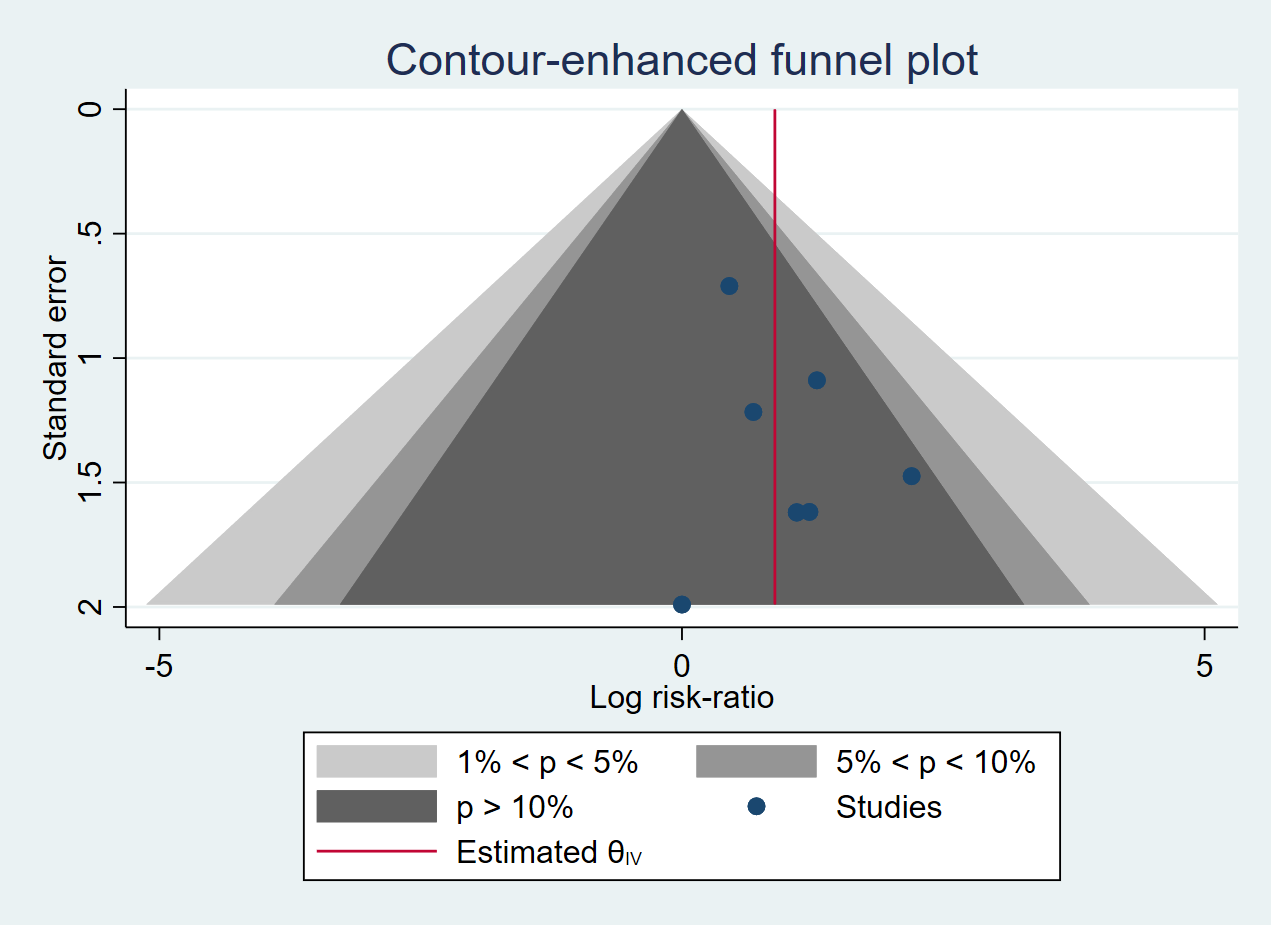


**Supplementary Figure 26. Contour-enhanced funnel plot of all complication rate.**


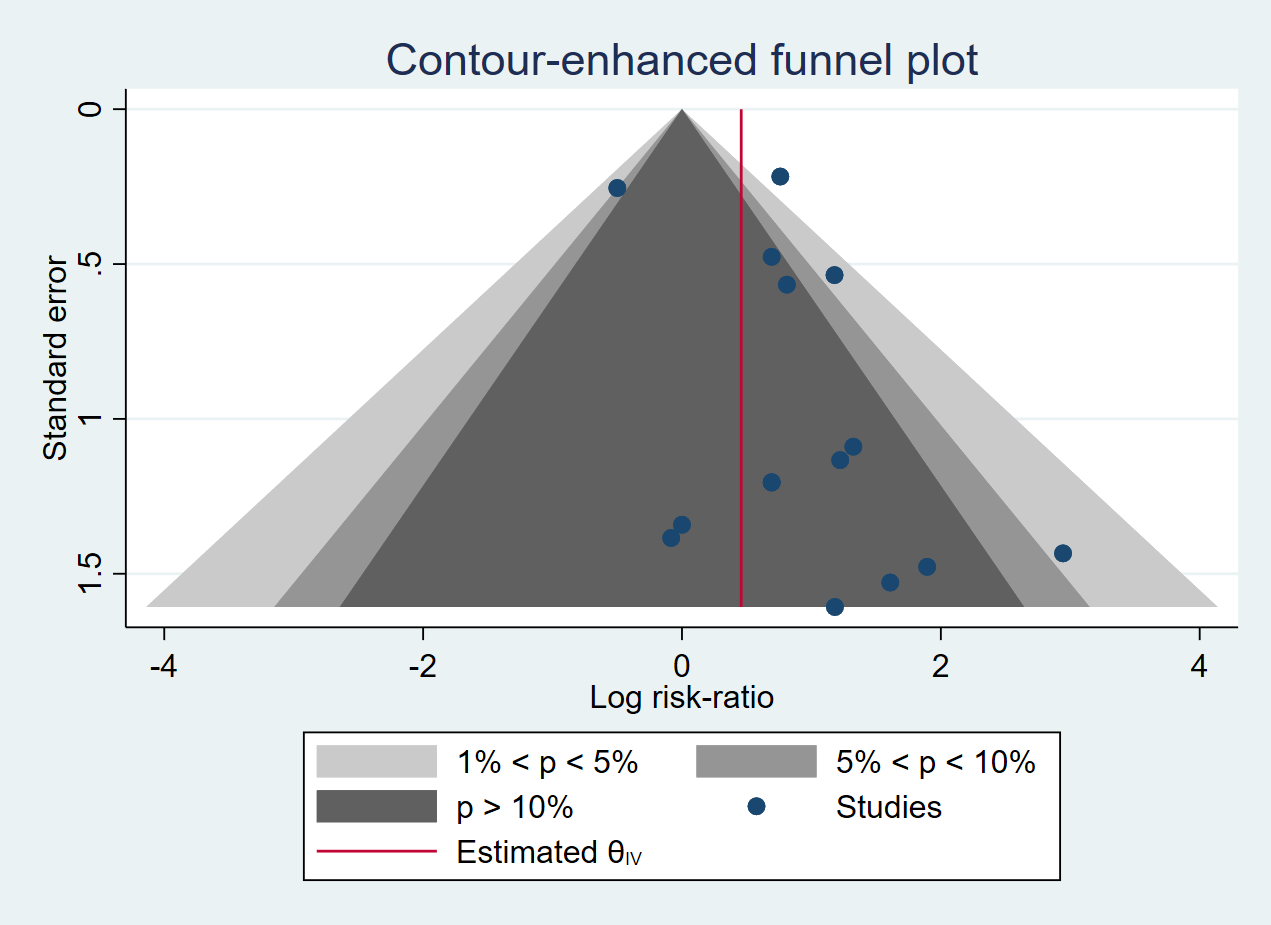


**Supplementary Figure 27. The forest plot regarding pulmonary embolism compared no tourniquet with tourniquet group.**


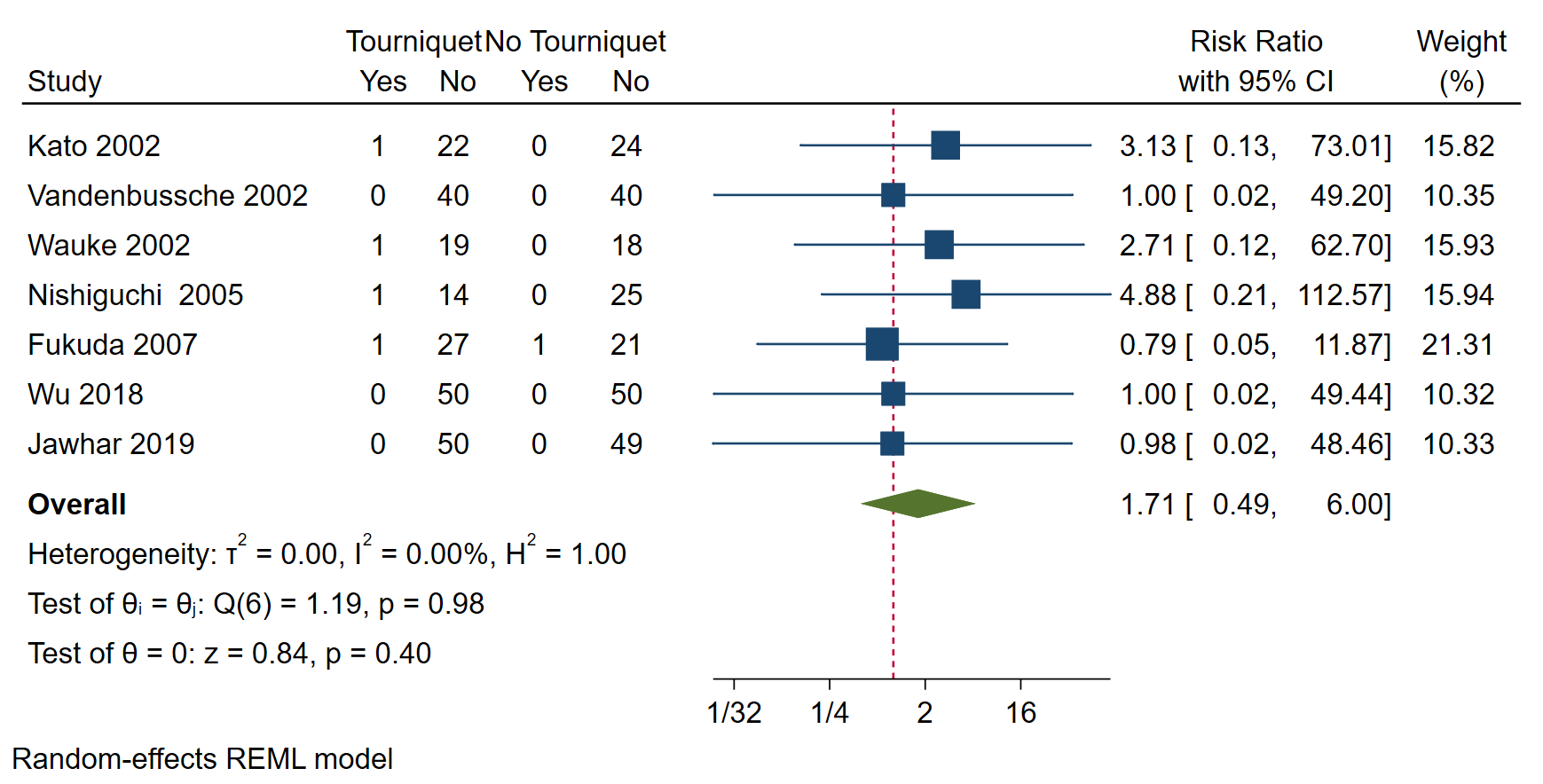


**Supplementary Figure 28. Contour-enhanced funnel plot of pulmonary embolism.**

**
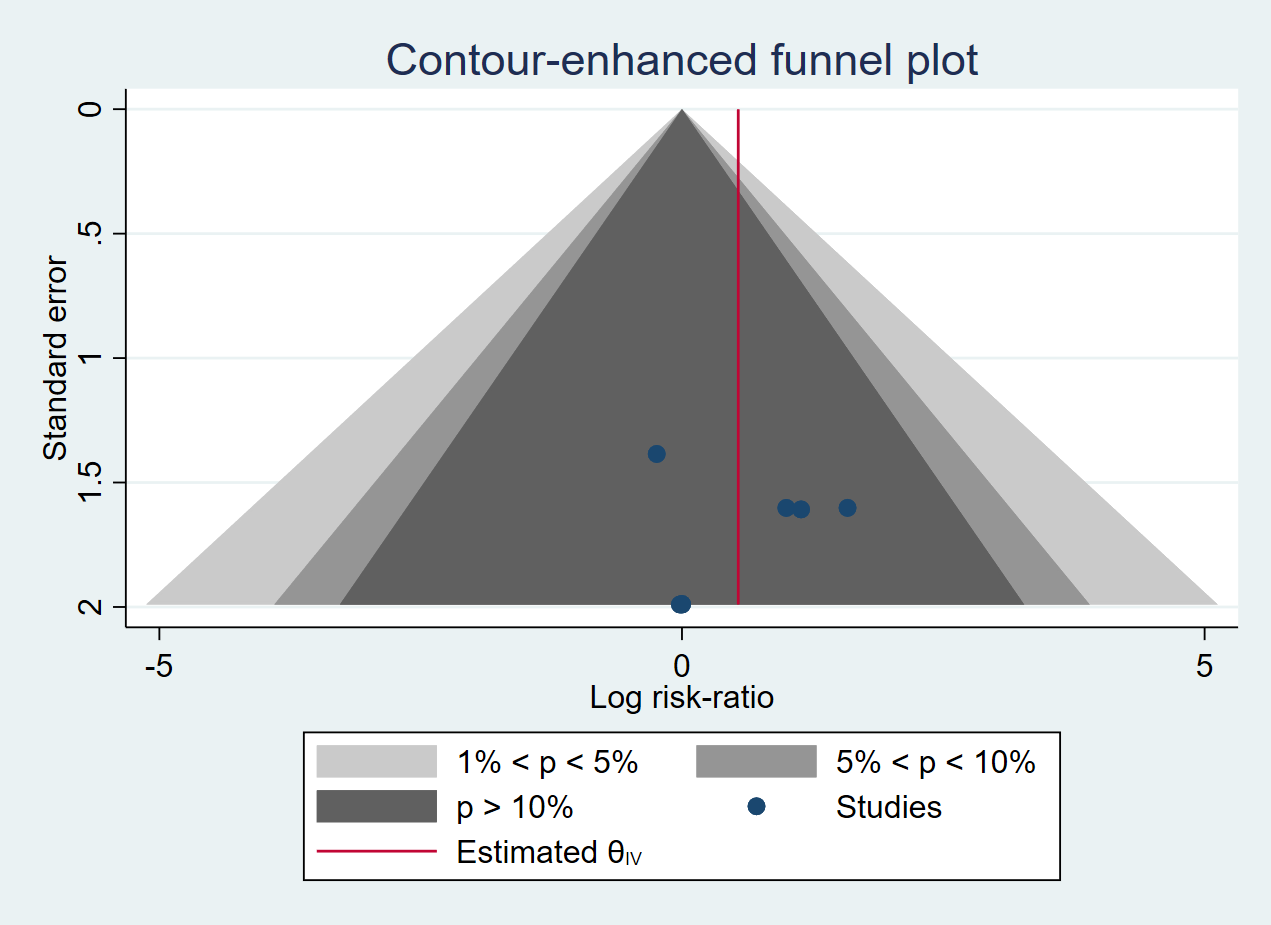
**

**Supplementary Table 1. Search strategy**

| **Database** | **Strategy** |
| --- | --- |
| **Pubmed** | (((((total knee arthroplasty[Title/Abstract]) OR (total knee replacement[Title/Abstract])) OR (TKA[Title/Abstract])) OR (TKR[Title/Abstract])) AND (tourniquet [MeSH Terms])) AND (randomized controlled trial[Publication Type]) |
| **the Cochrane Library** | MeSH descriptor: [Arthroplasty, Replacement, Knee] this term only  MeSH descriptor: [Tourniquets] this term only  in Trials |
| **Embase** | 'total knee arthroplasty':ab,ti AND tourniquet:ab,ti AND [randomized controlled trial]/lim |

**Supplementary Table 2. Inclusion/exclusion criteria of literature**

| **PICOS** | **Inclusion** | **Exclusion** |
| --- | --- | --- |
| P | 18 years of age or older who were scheduled for a primary total knee arthroplasty for end-stage osteoarthritis. | Rheumatoid arthritis, animals. |
| I | 1) tourniquet use during TKA;  2) No limit on sample size. | Revision procedures, previous knee surgery. |
| C | TKA without tourniquet. | Revision procedures, previous knee surgery. |
| O | 1) Primary outcomes including range of motion (ROM) and pain.  2) Secondary outcome included intraoperative blood loss, postoperative blood loss, measured total blood loss, calculated total blood loss, operation time, transfusion, deep vein thrombosis (DVT), superficial wound infection, and all complications (including DVT, infection, revision, wound erythema/ecchymosis and so on). | Relevant outcomes were missing. |
| S | RCT irrespective of blinding or arm. | 1) Articles without peer-reviewed or unpublished;  2) Studies that were repeatedly published or had qualitative outcomes;  3) Quasi-experimental studies, crossover, and observational studies. |

**Supplementary Table 3. Risk of bias table.**

| 1 | Random sequence generation (selection bias) |
| --- | --- |
| 2 | Allocation concealment (selection bias) |
| 3 | Blinding of participants and personnel (performance bias) |
| 4 | Blinding of outcome assessment (detection bias) |
| 5 | Incomplete outcome data (attrition bias) |
| 6 | Selective reporting (reporting bias) |
| 7 | Other bias |

**Supplementary Table 4. Characteristics of the Included Trials and Participants.**

| Number | Studies | Country | Cases/  Controls | Mean age,  case/control | Tourniquet pressure (mmHg) | Anesthesia | Tourniquet  duration | Drainage | Thromboprophylaxis | Follow-up |
| --- | --- | --- | --- | --- | --- | --- | --- | --- | --- | --- |
| 1 | Abdel-Salam 1995 | England | 40/40 | 72/74 | Twice SBP | General | Overall | Yes | Heparin | 2 years |
| 2 | Harvey 1997 | Canada | 16/28 | 72.4/73.4 | NS | NS | Part | Yes | Heparin, warfarin | 3 years |
|  |  |  | 36/28 | 68.3/73.4 | NS | NS | Overall | Yes | Heparin, warfarin | 3 years |
| 3 | Wakankar 1999 | England | 37/40 | 72.5/71.8 | Twice SBP | General | NS | Yes | Warfarin | 6 weeks |
| 4 | Aglietti 2000 | Italy | 10/10 | 70/68 | 608 | Regional | Part | Yes | NS | NS |
| 5 | Clarke 2001 | England | 10/10 | NS | SBP + 125 | General | NS | Yes | NS | NS |
|  |  |  | 10/10 | NS | SBP + 250 | General | NS | Yes | NS | NS |
| 6 | Tetro 2001 | Jamaica | 33/30 | 69.8/69.8 | SBP + 125 –  150 (max 300) | Hybrid | Part | Yes | Coumarins | 7 days |
| 7 | Kato 2002 | Japan | 22/24 | 65/63 | 350 | General | Part | NS | Heparin | NS |
| 8 | Vandenbussche 2002 |  | 40/40 | 72.5/68.5 | 350 | General | Overall | Yes | Heparin | 3 months |
| 9 | Wauke 2002 | France | 19/18 | NA | SBP + 100 | General | Part | NS | Heparin | 4 weeks |
| 10 | Katsumata 2005 | Japan | 25/25 | 67.19/65.36 | NS | General | NS | NS | NS | NS |
| 11 | Matziolis 2005 | Germany | 10/10 | 72.4/76.6 | 400 | Hybrid | NS | NS | NS | NS |
| 12 | Nishiguchi 2005 | Japan | 14/25 | 68/69 | NS | Hybrid | NS | NS | None | NS |
| 13 | Fukuda 2007 | Japan | 27/21 | 71.2/73.1 | 350 | Hybrid | Part | Yes | NS | 5 days |
| 14 | Kageyama 2007 | Japan | 12/12 | 73/76 | NS | Hybrid | NS | NS | Heparin | NS |
| 15 | Li 2008 | China | 30/30 | 71/70 | SBP + 100 | NS | Overall | None | Heparin | 7 days |
| 16 | Li 2009 | China | 40/40 | 71/70 | SBP + 100 | Hybrid | Overall | None | Heparin | 7 days |
| 17 | Yavarikia 2010 | Iran | 36/31 | 64/66 | 220–275 | Regional | Part | Yes | Heparin | NS |
|  |  |  | 29/31 | 68/66 | 220–275 | Regional | Overall | Yes | Heparin | NS |
| 18 | Zhang 2010 | China | 30/30 | 72/71 | SBP + 100 | NS | Overall | NS | Heparin | NS |
| 19 | Ledin 2012 | Sweden | 25/23 | 70/71 | 275 | Regional | NS | Yes | Heparin | 2 years |
| 20 | Tai 2012 | China | 36/36 | 72.1/71.5 | SBP + 100 | NS | Part | None | None | 4 days |
| 21 | Ejaz 2014 | Denmark | 33/31 | 68/68 | 250 | Regional | Overall | NS | None | 12 months |
| 22 | Liu 2014 | Australia | 10/10 | 67/70 | 300 | General | Overall | Yes | NS | 12 months |
| 23 | Dennis 2016 | USA | 28 | 62 | 250 | Hybrid | Part | Yes | NS | 3 months |
| 24 | Huang 2017 | China | 50/50 | 66.2/65.1 | SBP + 100 | General | Overall | Yes | Enoxaparin | 6 months |
| 25 | Liu 2017 | China | 52 | 67 | SBP + 125 | NS | Overall | Yes | NS | 90 days |
| 26 | Zhou 2017 | China | 72/68 | 66.8/69.1 | NS | General | NS | Yes | Rivaroxaban | 6 months |
| 27 | Alexandersson 2018 | Sweden | 38/43 | 68/ 69.7 | 300 | NS | NS | NS | Heparin | 3 months |
| 28 | Ozkunt 2018 | Turkey | 24/25 | 65.05 | NS | General | Overall | Yes | Heparin | 6 weeks |
|  |  |  | 20/25 | 65.05 | NS | General | Part | Yes | Heparin | 6 weeks |
| 29 | Wu 2018 | China | 50/50 | 67.58/68.06 | 250 | General | Part | Yes | Enoxaparin, rivaroxaban | 6 months |
| 30 | Goel 2019 | USA | 100/100 | 66/65.5 | 300 or 225 | Regional | Overall | NS | Aspirin | 8 months |
| 31 | Jawhar 2020 | Germany | 50/49 | 69.3/68.3 | 360 | NS | Overall | NS | NS | 6 months |
| 32 | Zhao 2020 | China | 60/60 | 65.01/64.53 | SBP + 100 | Regional | Overall | None | Enoxaparin | 3 months |
|  |  |  | 60/60 | 65.55/64.53 | SBP + 100 | Regional | Part | None | Enoxaparin | 3 months |
| 33 | Zeng 2021 | China | 50/50 | 68.44/68.00 | SBP + 100 | NS | NS | Yes | Heparin | 3 months |
|  |  |  | 50/50 | 68.66/68.00 | SBP + 100 | NS | NS | Yes | Heparin | 3 months |
